# Distinct molecular pathways leading to dosage-dependent temozolomide resistance in GBM stem cells

**DOI:** 10.1186/s12935-026-04213-6

**Published:** 2026-02-24

**Authors:** Hany E. Marei, Giacomo Pozzoli, Alice Gaiba, Michele Sonnessa, Carlo Cenciarelli

**Affiliations:** 1 Mansoura University, Department of Cytology and Histology at the Faculty of Veterinary Medicine, Mansoura, 35116 Egypt; 2 Pharmacology Section, Department of Health Care Surveillance and Bioethics, Università Cattolica del Sacro Cuore, Rome, Italy; 3 Fondazione Policlinico Universitario A. Gemelli IRCCS, Rome, Italy; 4 Institute of Translational Pharmacology-CNR, Rome, Italy; 5 Bio-Fab Research Srl, Rome, 00161 Italy

**Keywords:** Glioblastoma (GBM), Glioblastoma stem cells (GSC), Temozolomide (TMZ) resistance, Stemness, Stem cells plasticity, Anti-apoptotic molecules, Neuroactive ligand-receptor interactions, Perivascular niche

## Abstract

**Background:**

Glioblastoma (GBM) is the most aggressive primary brain tumor in adults, with a median survival of around 15 months despite complete therapy. A significant contributor to recurrence is the enduring presence of GBM stem cells (GSC), which exhibit remarkable self-renewal, adaptability, and resistance to treatment measures.

**Methods:**

Patient-derived cancer stem cells (GSC) were continuously exposed to temozolomide (TMZ) in vitro to create a model for investigating chemotherapy resistance. Transcriptomic profiling was conducted to investigate the molecular pathways associated with resistance, with Western blotting used to confirm the findings from RNA sequencing. The analyses focused on signaling pathways related to neurosynaptic transmission, stemness, pro-survival adaptability, ECM remodeling, and DNA repair.

**Results:**

A convergent multi-pathway adaptation was noted in GSC treated with various dosages of TMZ. A transcriptomic analysis indicated that cells exposed to high-dose TMZ (TMZ-hc) displayed a specific activation of a neuroactive, synaptic-like expression program. This program included genes associated with neurotransmitter receptors as well as voltage-gated calcium and potassium channels, while simultaneously suppressing DNA mismatch repair mechanisms and negative feedback regulators. In contrast, an alternative resistance pathway was discovered in cells treated with low-dose TMZ (TMZ-Lc), which promoted a niche-dependent, dormant state marked by the expression of vascular mimicry markers and remodeling of the extracellular matrix. In addition, the protein levels of Survivin, Bcl-2, and Notch1 signaling were significantly elevated in TMZ-hc compared to TMZ-Lc and control cells.

**Conclusions:**

Our research underscores the translational significance of investigating GSC-specific resistance mechanisms, since GSC are recognized as the primary drivers of patient recurrence. Understanding the molecular mechanisms that enable TMZ resistance is crucial to developing new therapeutic options.

**Graphical Abstract:**

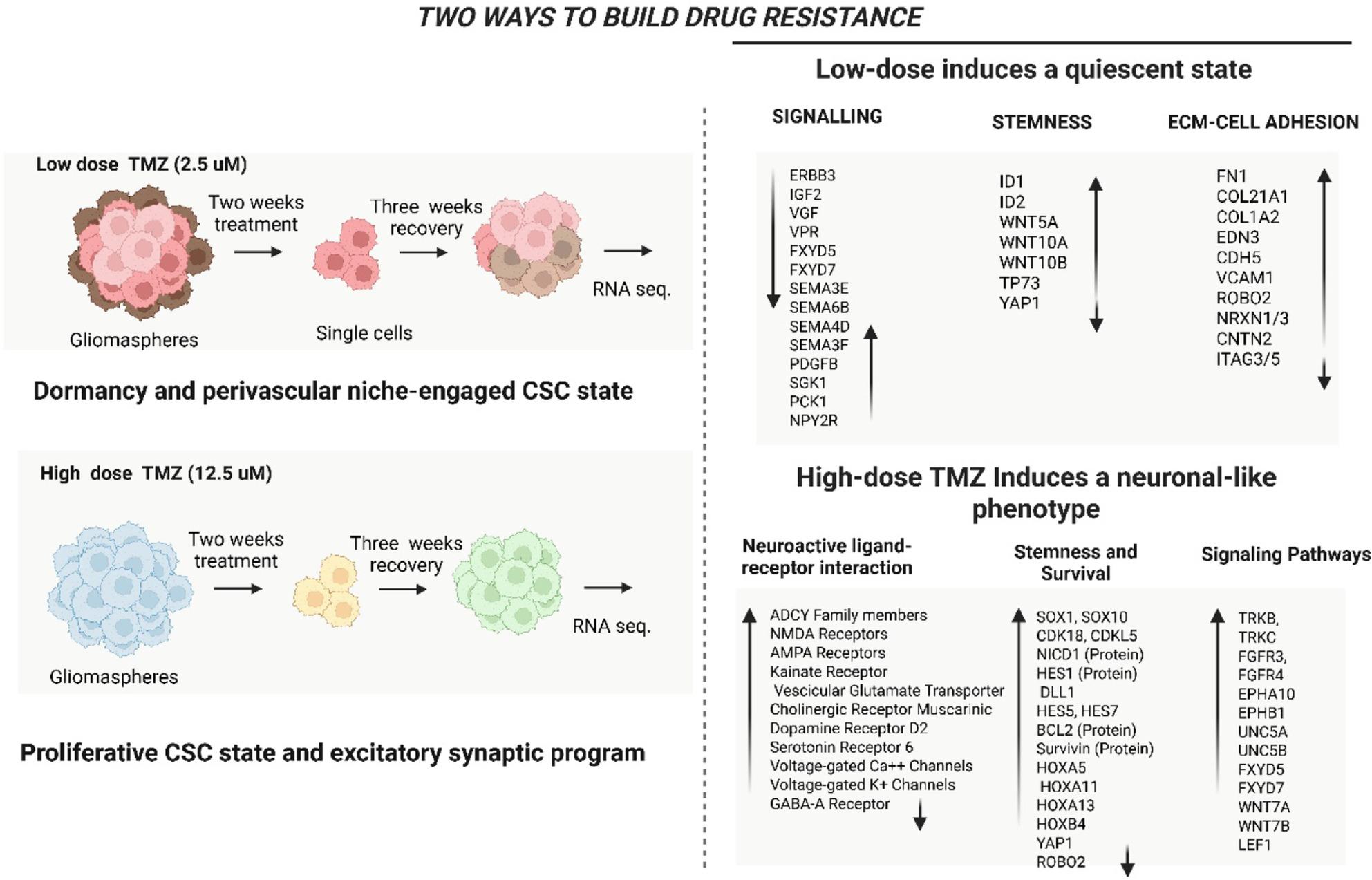

**Supplementary Information:**

The online version contains supplementary material available at 10.1186/s12935-026-04213-6.

## Introduction

GBM (GBM) is one of the most challenging diseases to treat in oncology because of its diffuse infiltrative nature and nearly ubiquitous pattern of recurrence. Neurosurgery and perioperative strategies have seen significant improvements in recent years. However, the current standard of care—maximal safe resection followed by radiotherapy combined with concomitant and adjuvant temozolomide (TMZ) administration—only slightly improves clinical outcomes. The typical survival is 14–16 months, and less than 30% of patients are alive two years after diagnosis [1, 2]. TMZ is a cytotoxic agent that can induce cell death by methylating the O6 position of guanine residues. The DNA mismatch repair (MMR) pathway can detect this damage and repair the breaks in the DNA strand. However, a deficiency in MMR may result in ineffective repair cycles, leading to cancer and genomic instability [3]. Moreover, the effectiveness of TMZ is significantly hampered by both intrinsic and acquired resistance mechanisms, which can cause rapid disease progression and a poor prognosis [4, 5]. A subpopulation of tumor-initiating GBM stem cells (GSC) forms the core of this resistance. These cells demonstrate multipotency, retain their self-renewal abilities, and are characterized by enhanced DNA repair mechanisms and increased resistance to therapeutic damage, all of which promote recurrence and tumor heterogeneity [6, 7]. Extensive research using lineage tracing and xenograft models has demonstrated that GSC can resist TMZ treatment and subsequently regenerate tumors, highlighting their crucial role in resistance and recurrence [8, 9]. 

The molecular pathways that support the viability of GSC during TMZ stress are intricate and involve developmental pathways, transcriptional regulators, and adaptation mechanisms to the tumoral microenvironment. The Notch1 signaling pathway, often reactivated in GBM, plays a crucial role in the regulation of stem cell fate. The activation of Notch1 preserves stemness, prevents differentiation, and provides resilience against genotoxic stress, making it an intriguing yet intricate therapeutic target [10, 11]. Additionally, the Stat3 pathway, which is activated by cytokines and growth factors present in the GBM microenvironment, may contribute to the failure of therapy. Stat3 regulates growth, immune evasion, and cell survival, and has been closely associated with the maintenance of GSC in a drug-tolerant condition [12, 13]. The direct inhibition of apoptosis is another hallmark of chemotherapy resistance. Bcl-2 and Survivin (*BIRC5*), which belong to the inhibitors of apoptosis (IAP) family, are among the most significantly overexpressed proteins in GBM. By hindering caspase activation, they prevent cell apoptosis. Thus, the overexpression of Survivin is linked to a poor prognosis, while its inhibition restores sensitivity to TMZ in experimental animal models [14, 15]. 

Ultimately, GBM cells are dependent on the unique brain environment to maintain their homeostasis. Recent research suggests that GSC can integrate into neural networks and utilize neuroactive ligand-receptor signaling to promote proliferation and evade therapeutic interventions. Neurotransmitter receptors, ion channels, and G-protein-coupled receptors (GPCRs) found on GSC3 line facilitate synaptic communication and boost calcium/cAMP-dependent pro-survival signaling [16, 17]. Interestingly, these neuroglial connections not only support tumor proliferation but also open up new therapeutic avenues that are distinct from conventional DNA repair mechanisms. In this research, we suggest that the resistance of GSC3 line to TMZ may not stem from isolated molecular modifications, but rather from the simultaneous activation of various adaptive pathways, including Notch1, Bcl-2, Survivin, Erk1/2, Akt1, and neuroactive signaling.

Although significant effort has been put into exploring the ability of glioblastoma stem cells to adapt dynamically to temozolomide (TMZ), therapeutic resistance is frequently considered to be either “sensitive” (responding positively to therapy) or “resistant” (not responding). However, there is often little attention paid to how the magnitude of chemotherapy administration affects the types of adaptive states present in GSC. For example, within both clinical settings and within the tumor’s environment, the amount of exposure to TMZ will vary significantly based on a large number of factors. Some of these factors may include differences in dosing regimens, drug penetration capabilities, and the local structural architecture associated with each tumor, which creates different levels of stress specific to each area within the tumor. Based on these observations, we hypothesized that (i) the transcriptional and signaling programs activated in response to TMZ by GSC3 line at low doses would differ from those induced at high doses; and (ii) the activation of low-dose TMZ exposure would promote the development of mechanisms of stress tolerance and maintenance of quiescent and place-based survival, while activation of high-dose TMZ exposure would result in major changes in the activation of DNA repair processes, oncogenic signaling pathways, and lineage-associated genes. Therefore, to fill this knowledge gap, the objective of the current study was to directly compare the different adaptive transcriptional profiles exhibited by patient-derived GSC3 line exposed to both low- and high-dose TMZ to specifically identify differences in the dose-dependent resistance profiles that would otherwise remain undifferentiated if only the response to TMZ treatment was evaluated uniformly across GSC3 line. The identification of these different adaptive states will allow for a better understanding of how the intensity of therapeutic pressure affects the plasticity and resistance pathways associated with GSC3 line.

## Materials and methods

### GBM stem cells culture

GBM non-necrotic samples were obtained from patients undergoing neurosurgery who had not received prior chemotherapy or radiotherapy. The tumors were dissected and digested in a papain solution (Worthington Biochemical, Lakewood, NJ, USA) and banked in accordance with research ethics board approval from the Institute of Neurosurgery, Catholic University of the Sacred Heart of Rome, Italy (Prot. RBAP10KJC5). Patients gave informed consent before surgery [18]. Furthermore, all applicable biosafety regulations were followed during tissue collection.

Glioblastoma stem cells (GSC3 line) were cultivated as previously described [18–20]. In summary, cells grow spontaneously in suspension as neurospheres in the presence of human recombinant EGF (20 ng/mL) and human recombinant bFGF (10 ng/mL; PeproTech, Rocky Hill, NJ, USA) in serum-free medium DMEM/F12 (1:1) (Invitrogen, Thermo Fisher). Mid-sized neurospheres were enzymatically dissociated using Accutase (Merck Millipore, Darmstadt, Germany) for 1–2 min at 37 °C and replated as single cells to promote healthy cell proliferation. This formulation retained and enhanced stem-like traits, such as self-renewal and multipotency, while reducing the rate of spontaneous differentiation. To establish a model of acquired TMZ resistance, GSC3 line was exposed to escalating doses of temozolomide (TMZ; Sigma-Aldrich) over a period of 2 weeks, starting with sub-lethal doses (2.5 µM) and progressing to high doses (12.5 µM) [21, 22]. This extended exposure resulted in the death of the majority of the GSC3. Following the removal of the TMZ-medium, the surviving cells, allowed to recover for three weeks, were classified as TMZ-hc and TMZ-Lc, respectively. Parallel cultures of the same parental GSC3 line, maintained in a vehicle (DMSO, 0.1%) without TMZ exposure, served as matched controls (CTRL).

### Western blots

Whole-cell protein extracts are obtained from TMZ-hc GSC3 line and control cells cultured logarithmically using RIPA buffer containing protease and phosphatase inhibitor cocktails (Roche Diagnostics). Protein extract concentrations were measured using the Bio-Rad protein Assay (Bio-Rad, Munich, Germany). 30 µg of protein were loaded onto Bolt 4–12% Bis-Tris gels (Invitrogen), subsequently transferred to Hybond-P Extra membrane (Amersham Biosciences, GE Healthcare), and then blocked with 5% non-fat milk. The filters were subjected to immunoblotting with the following primary antibodies: rabbit anti-EGFR, rabbit anti-cleaved Notch1 (NICD1, Val 1744), rabbit anti-HES-1, anti-T202/Y204-ERK1/2, anti-ERK1/2, rabbit anti-S473-AKT1, and rabbit anti-AKT1 (purchased from Cell Signaling Technology, USA), mouse anti-BCL2 (Dako Denmark), rabbit anti-Survivin (Abcam, Cambridge, UK), and mouse anti-β-actin (SIGMA-Aldrich). Following three washes with TBS-Tween buffer, the immunoreactive proteins were visualized using rabbit anti-mouse and goat anti-rabbit horseradish peroxidase-conjugated secondary antibodies specific to the respective primary antibodies (Jackson Immunoresearch, West Grove, PA). Images acquisition was performed using ChemiDoc imaging systems (Bio-Rad-USA).

Using densitometric analysis, the intensity of the bands was quantified, and this data was normalized to the β-actin. The determined values were later expressed as a percentage of the control value. The densitometric analyses of bands normalized to β-actin protein levels were performed using the ImageMasterR VDS and the Imagesystem software package (Amersham‐Pharmacia Biotech). Data were analyzed by one‐way ANOVA, followed by post hoc Newman‐Keul test for multiple comparisons among group means, using a PrismTM software (GraphPad), and differences were considered statistically significant if *P* < .05. All results are presented as the mean ± SEM of at least two different experiments (*n* = 2) performed in duplicate, unless otherwise specified.

### Bioinformatic integration and transcriptomic profiling

We used the RNeasy Mini Kit (Qiagen) to obtain the total RNA, following the manufacturer’s instructions. The Agilent 2100 Bioanalyzer verified the RNA’s integrity, and samples exhibiting a RIN over 8.0 were selected for sequencing. The VAHTS Universal v10 RNA-Seq kit with VAHTS mRNA Capture Beads 2.0 (Vazyme, Nanjing, PRC) was used for library preparation according to the manufacturer’s instructions, and the library was sequenced on an Illumina NovaSeq X-Plus platform (Illumina, San Diego, CA) to obtain 150 bp paired-end reads. The quality of the raw readings was evaluated using FastQC v0.12.1. Cutadapt v3.4 was used to trim them down, and the STAR aligner v2.7.10b was used to align them to the human genome Reference GRCh38.p14 (hg38) [23]. We utilized the edgeR v4.6.0 [x] in R version 4.4.3 to examine gene expression differences between groups (CTRL, TMZ-hc, and TMZ-Lc) [24]. The thresholds were |log2 fold change| > 1 and an adjusted p-value (FDR) < 0.05.

Differentially expressed genes (DEGs) were subjected to pathway enrichment analysis using the Kyoto Encyclopedia of Genes and Genomes (KEGG) and Gene Ontology (GO) databases, with clusterProfiler v4.16.0 utilized for visualization [25]. We used over-representation analysis (ORA) with FDR control to identify changes in biological pathways that co-occurred. To enhance translational applicability, we integrated our lists of differentially expressed genes (DEGs) with publicly accessible datasets, including The Cancer Genome Atlas GBM cohort (TCGA-GBM), to assess clinical correlation and prognostic significance [26]. 

RNA-seq analysis utilized independent biological replicates [*n* = 3] of GSC3 line representing each of the experimental conditions: Ctrl, TMZ-hc, and TMZ-Lc. All samples underwent the same library preparation and sequencing.

## Results and discussion

### The transcriptome landscape of high-dose TMZ reveals a phenotype-like synapse and failure to repair DNA

**Fig. 1:**
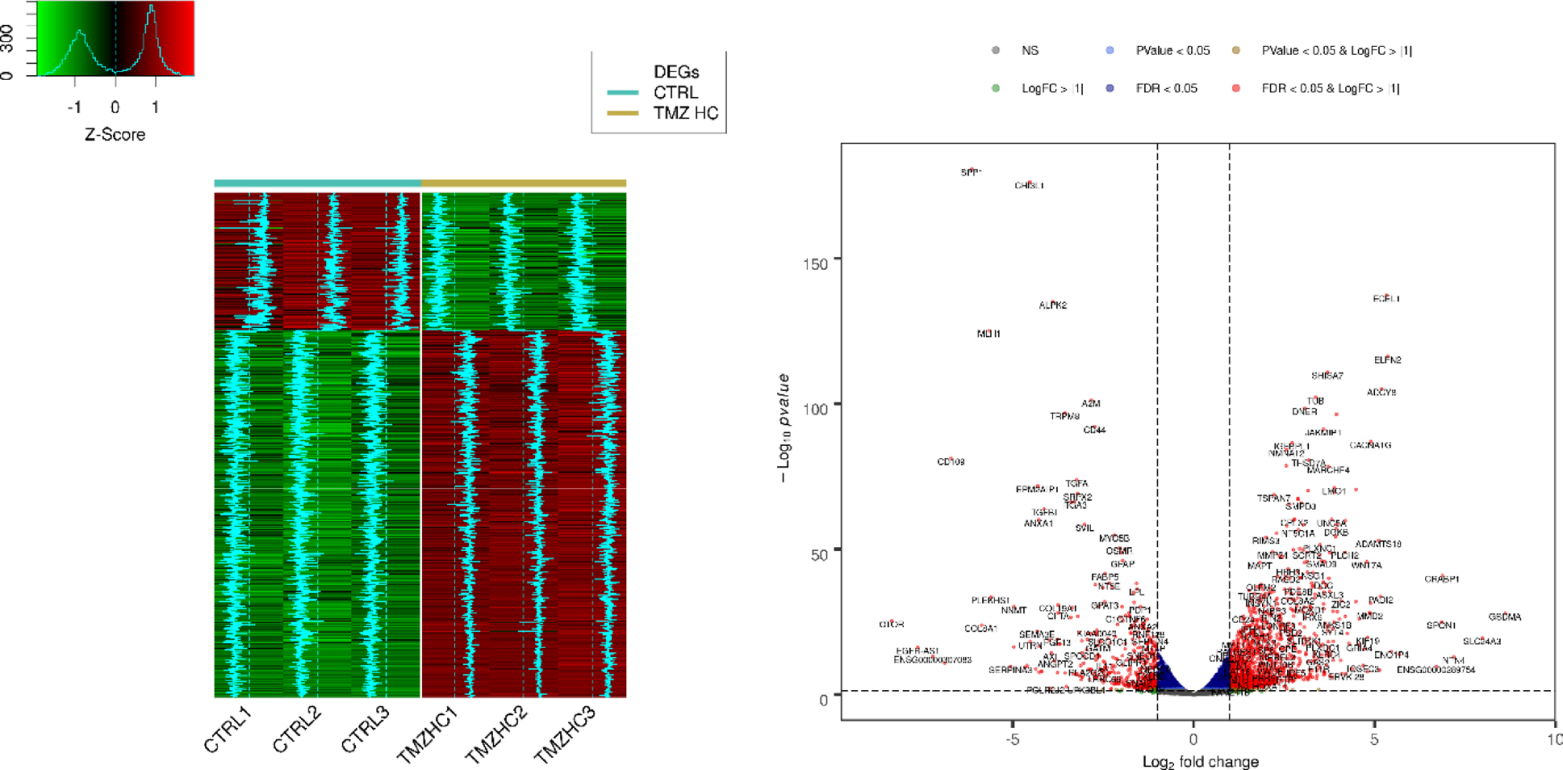
Transcriptomic analysis with statistically significant changes in expression levels between TMZ-HC and control GSC3. The left panel shows a heatmap plot of differentially expressed genes (DEGs) patterns (|log2FC| > 1 and FDR < 0.05) across all samples. Trends of the DEGs are shown in light blue, relative to the mean value. The right panel shows a volcano plot of the differential expression (DE) analysis for all genes identified using RNA-seq, with up- and down-regulated genes highlighted in red dots. The scatterplot is color-coded based on different combinations of FDR and |log2FC|. The data used were obtained from patient-derived GSC3 line, with three biological replicates performed for each of the conditions

Through bulk RNA-seq analysis, we identified differences between TMZ-hc and DMSO-treated GSC3 line (Ctrl), as shown in the heatmap (Fig. 1, left panel), which displays distinct gene expression patterns unique to each cell group. The significantly upregulated and downregulated genes in the two populations were also illustrated through a volcano plot analysis **(**Fig. 1, right panel). RNA sequencing revealed that, among the 1,670 differentially expressed genes in TMZ-hc-treated GSC3 line compared to controls, 1,214 (72.7%) were upregulated. In contrast, 456 (27.3%) were downregulated (**Fig. **S1).

**Fig. 2:**
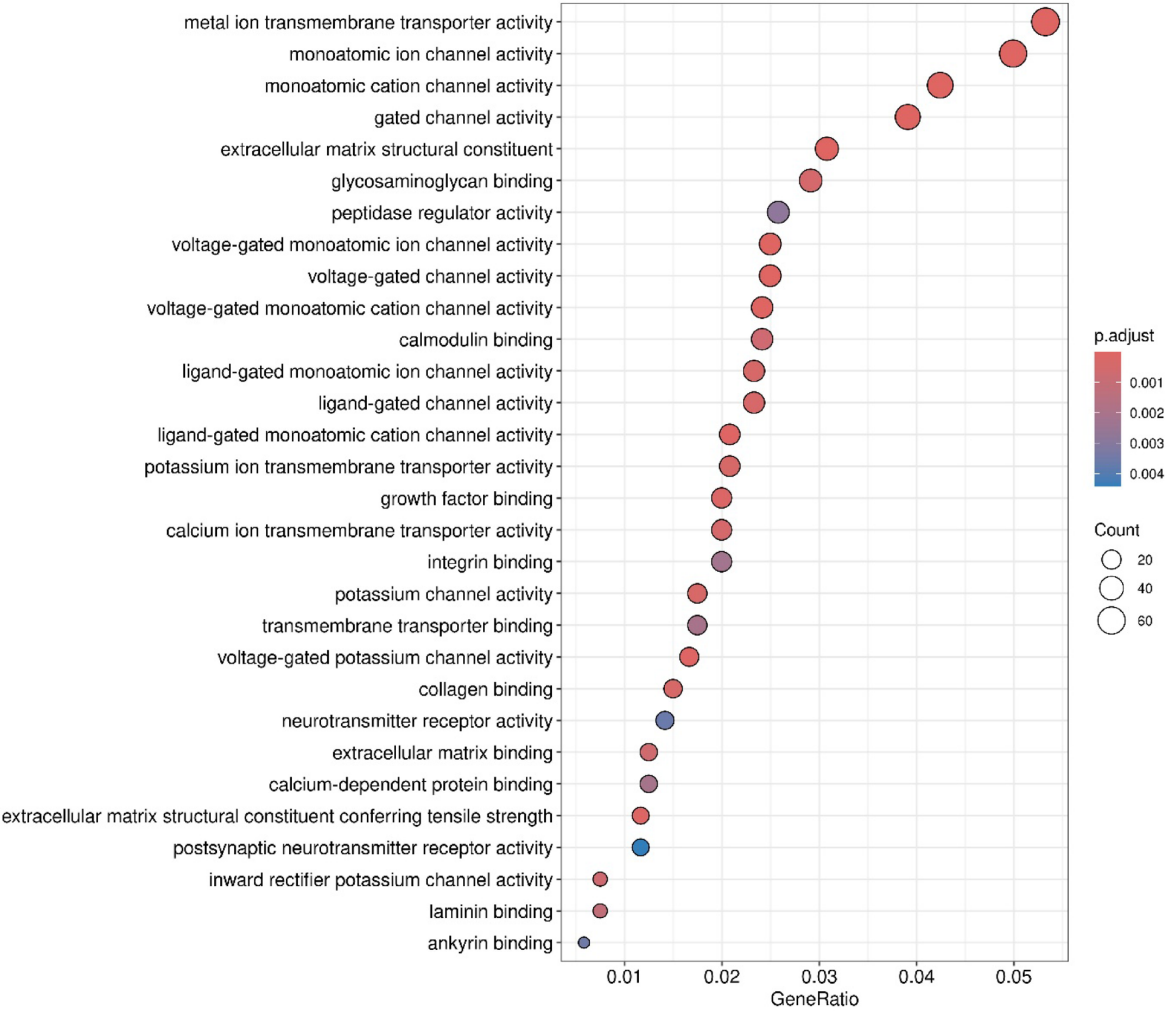
Gene Ontology (GO) molecular function (MF) enrichment analysis of DEGs between TMZ-HC and control GSC3. Dot plots of over-representation analysis (ORA) results using GO (FDR < 0.05) in the molecular function (MF) category (biochemical activity of the gene product) to interpret the function of DEGs obtained from RNA-Seq analysis results between TMZ-HC and Control (CTRL) samples. The size of the dots represents the number of genes in the significant DE gene list associated with the GO term, and the color of the dots indicates the P-adjusted values (BH)

**Fig. 3:**
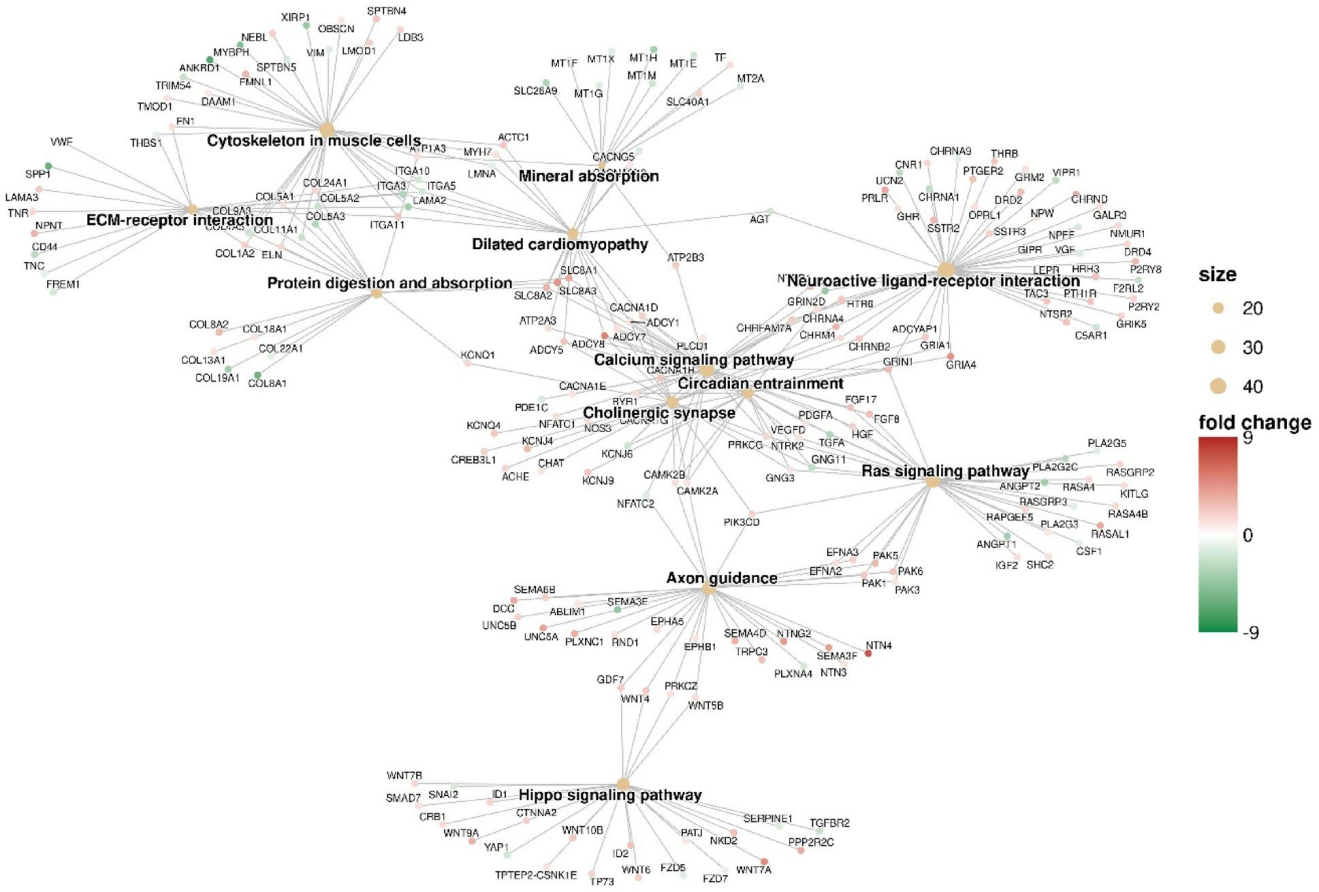
KEGG pathway enrichment network of DEGs between TMZ-HC and control GSC3. A network plot has been created to illustrate the ORA results from KEGG pathways (FDR < 0.05) for genes that are DE between the TMZ-HC and CTRL. Each node represents a KEGG pathway that is enriched based on DEGs, with the node size corresponding to the number of DEGs associated with a pathway. The colour of each node indicates the degree of change (increase or decrease) in gene expression that occurred between the TMZ-HC and CTRL groups. Fold change represents the differences in fold change between the TMZ-HC group and the control GSC3 line (CTRL)

**(Fig. **S1). A | log 2 FC | greater than 1 and a false discovery rate (FDR) below 0.05 were set as thresholds for identifying statistically significant differentially expressed RNAs. Figure 2 presents a Gene Ontology analysis within the molecular function (GO-MF) category, aimed at interpreting the functions of differentially expressed genes from RNA-Seq results in TMZ-hc (12.5 µM) *vs* Ctrl. The most notable process was the initiation of a highly specialized program focused on neuroactive ligand–receptor interactions signaling (Figs. 2 and 3). A significant upregulation involved key members belonging to the neuroactive ligand- receptor signaling, such as the adenylyl cyclase family (*ADCY8*, *ADCY5*, *ADCY7*, *ADCY1*; with a log2-fold change of + 5.19, + 2.51, + 1.33 and + 1.20 vs. controls, respectively), N-methyl-D-aspartate (NMDA) receptor subunits (*GRIN1*, *GRID2*; with a log2-fold change of + 2.81 and + 1.38 vs. controls, respectively), the ionotropic glutamate receptor (iGluR) subunit *GRIK5* (log2-fold change of + 1.88 vs. controls), the dopaminergic receptor *DRD2* (log2-fold change of + 2.76 vs controls), serotonergic receptor *HTR6* (log2-fold change of + 2.42 vs controls), the cholinergic muscarinic receptor *CHRM4* (log2-fold change of + 1.49 vs. controls), and several voltage-gated Ca^2+^ channel subunits (*CACNA1G*, *CACNA1H*, *CACNA1D*; with a log2-fold change of + 4.81, + 2.28 and + 1.98 vs. controls, respectively). Additionally, voltage-gated K^+^ channels, such as *KCNQ4* (log2-fold change of + 2.38 compared to controls), *KCNB1* (log2-fold change of + 2.75 compared to controls), and *KCNN1* (log2-fold change of + 3.85 compared to controls), were involved. This transcriptional reprogramming indicates that resistant GSCs exhibit an excitatory synapse-like characteristic, promoting calcium and cAMP signaling, which supports cell survival. The coordinate transcriptomic enrichment of glutamate receptors, ion channels, and other synaptic component genes is what has been defined as “synaptic-like transcriptional program. It has not been established that this phenotype correlates directly to any actual functional activation of synapses or neurotransmitter release. The increase in neuroactive ligand-receptor interactions, genes for ion channels, and synaptic scaffolding molecules observed in TMZ-hc GSC3 indicates an apparent synaptic-like transcriptional program but does not provide any evidence that they undergo functional differentiation or participate in signaling. This is the first study to classify these findings solely on the basis of bulk RNA-sequence analyses; therefore, the results should be used as a starting point for future studies. Before confirming that the changes identified here reflect real synaptic function and altered neural excitability, these transcriptional changes must be validated using functional techniques such as electrophysiological recordings, calcium imaging, and neurotransmitter release assays. Therefore, the term “synaptic-like” is applied here to indicate that coordinated expression of several genes occurs together rather than that they demonstrate confirmed biological activity within the central nervous system.

On the other hand, there was a significant downregulation of *MLH1* (log2-fold change of -5.64 vs. controls), the more common deficiency component of a system of seven DNA mismatch repair (dMMR) proteins that cooperate in successive steps to initiate DNA mismatch repair. This was consistent with the established mechanism of TMZ resistance, which involves a decrease in MMR activity. These traits hypothetically place resistant GSC on a dual axis of survival, involving neuroactive, synaptic-like transmission and the suppression of DNA repair–dependent apoptosis.

A notable increase in *SOX1* and *SOX10* (log2-fold change of + 1.17 and + 3.34, respectively, compared to controls) and cell cycle-dependent kinase 18 (*CDK18*, log2-fold change of + 1.69 compared to controls) as well as cell cycle-dependent kinase-like 5 (*CDKL5*, log2-fold change of + 1.64 compared to controls) enhances the stemness characteristics of these cells.

Likewise, Wnt signaling is crucial for regulating stem cell self-renewal, cell proliferation, and determining cell fate in neural stem cells. Definitive evidence has shown that the hyperactivation of Wnt signaling is linked to malignant transformation and the development of brain tumors. Three distinct Wnt signaling pathways have been identified: the canonical Wnt/β-catenin pathway, the non-canonical planar cell polarity pathway, and the non-canonical Wnt/Ca^2+^ pathway. The role of one or both Wnt pathways (canonical and non-canonical) in the onset and progression of GBM remains uncertain [27]. 

Figure 3 displays a significant differential expression of various Wnt family **(**Fig. 3**)** members involved in Hippo signaling **(**Fig. 3**)** in GSC3 line exposed to a high dose of TMZ. This includes *WNT7A/B* (log2-fold change of + 4.78, + 1.63, respectively), *WNT5B* (log2-fold change of + 1.45), *WNT9A* (log2-fold change of + 3.32), *WNT10B* (log2-fold change of + 2.26), *WNT4* (log2-fold change of + 2.30), and *WNT6* (log2-fold change of + 2.07).^28^

The transcriptomic analysis of GSC3 line subjected to TMZ-hc revealed significant alterations in ATP-binding cassette (ABC) transporters. We specifically observed the upregulation of *ABCC8* and *ABCG4* and the downregulation of *ABCC3* and *ABCA13*. *ABCC8* (*SUR1*), which encodes the sulfonylurea receptor 1, a regulatory subunit of ATP-sensitive K⁺ channels, corroborates previous research demonstrating the elevation of *SUR1* in high-grade gliomas and its involvement in glioma proliferation and edema [29, 30]. The increase in *ABCC8* (log2-fold change of + 2.73) implies that survival pathways related to ion channels may be enhanced in GSC3 line under TMZ-induced stress. Similarly, *ABCG4*, a member of the G-subfamily of ABC transporters, primarily responsible for cholesterol and lipid efflux in the central nervous system, was elevated after TMZ-hc (log2-fold change of + 2.16). The role of ABCG transporters in gliomas remains unclear. Still, they are known to play a crucial role in multidrug resistance by facilitating the efflux of xenobiotics and chemotherapeutics from cells [31]. This means that overexpressing *ABCG4* may help cells become resistant to TMZ by protecting them through efflux. The *ABCG4* gene was shown to have increased transcription levels compared to those of other members of the ABC transporter family. No studies thus far show a clear function of ABCG4 pertaining to efflux or resistance against temozolomide in glioblastoma.

Regardless, ABCG4 is part of the G-Subfamily of ATP-Binding Cassette Transporters, which consists of ABC Transporters that transport lipids, as well as others that transport xenobiotics in certain contexts. The upregulation of *ABCG4* in TMZ-hc GSC3 indicates a possible function for *ABCG4* in mediating adaptational cellular responses to chemotherapy stress, instead of the confirmed functions of ABCG4 as efflux pumps for TMZ.

Conversely, *ABCC3* (*MRP3*) showed significant downregulation (log2-fold change of -2.63). ABCC3 acts as an efflux pump for organic anions and drug metabolites, and it has been associated with resistance mechanisms in various types of cancer [31]. The decrease in TMZ-hc-treated GSC3 line indicates a reduced detoxification ability, potentially making the cells more vulnerable to TMZ-induced DNA damage.

Ultimately, *ABCA13*, recognized as the largest ABC transporter identified to date, showed a significant downregulation (log2-fold change of -3.00). Although there is limited characterization of *ABCA13* in gliomas, its overexpression has been linked to aggressive phenotypes and poor therapeutic responses in various other cancers [32]. Inhibiting this activity under TMZ-hc may disrupt lipid transport and efflux-mediated survival pathways, suggesting increased vulnerability in GSC3 line exposed to TMZ.

It has been illustrated that neural tissue and microglia express high levels of ABCG4 in conditions affecting cholesterol and lipid homeostasis. Currently, there’s very little information available as to how this protein will affect cancer pathology, however there is a strong association with transcriptionally-induced ABCG family members (of which ABCG4 is included), when it comes to how cancers establish their resistance to chemotherapeutic agents based upon stress adaptation [31–34]. 

All of these data show that temozolomide dose intensity drives GSC3 to different transcriptional states through different levels of activity in DNA repair pathways, survival signaling and neuroactive gene expression programs. These changes represent distinct adaptive responses and not a single resistance phenotype.

### Low-Dose TMZ induces a quiescent, Niche-Engaged stem cell state

**Fig. 4:**
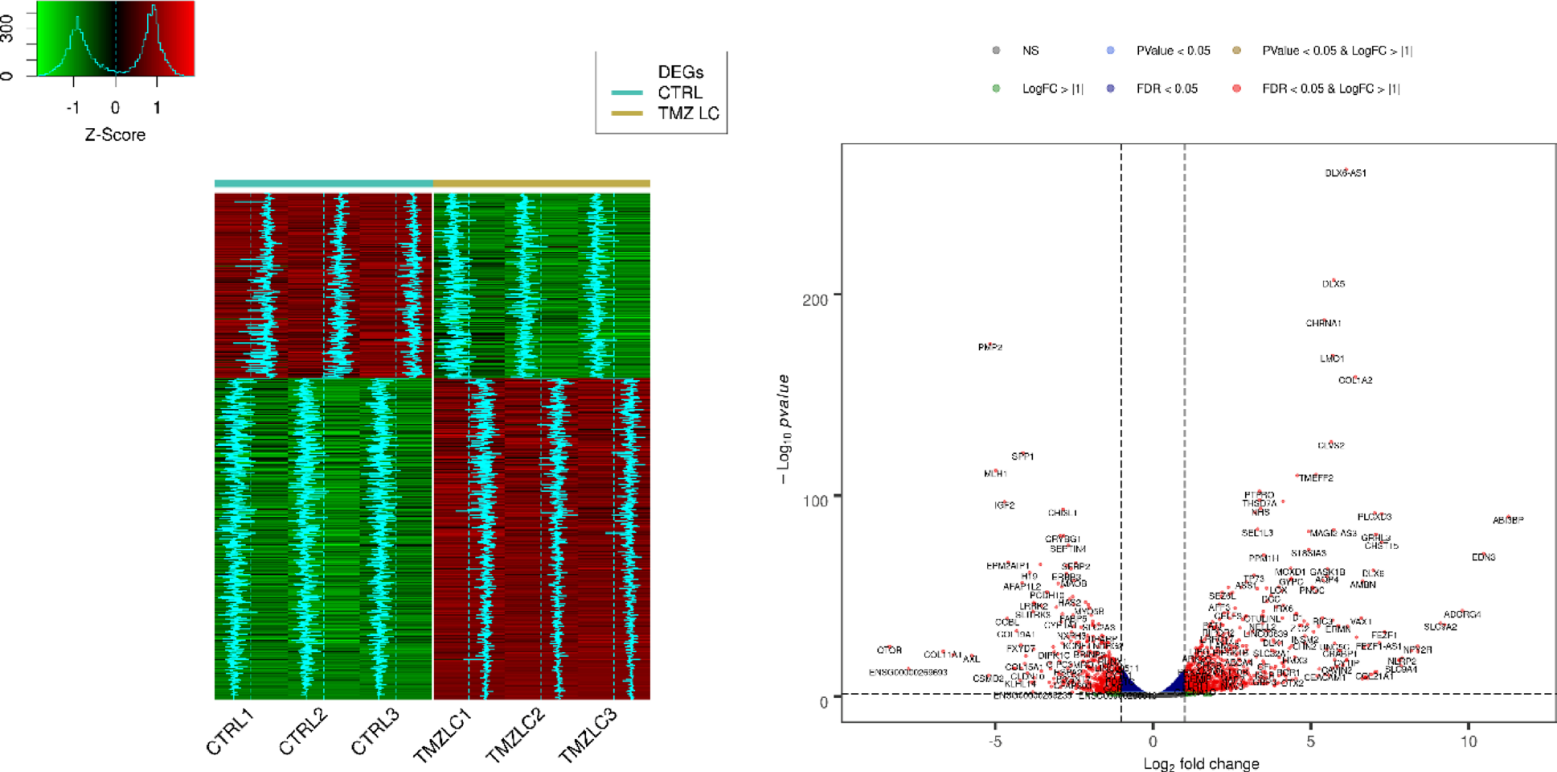
Transcriptomic differences between TMZ-LC and control GSC3. The left side of the panel contains a heatmap that illustrates the differential gene expression profiles (DEGs) observed when comparing the conditions of TMZ-Lc vs. CTRL. The DEGs that are shown meet the criteria of |log2FC| > 1 and false discovery rate (FDR) < 0.05. The heatmap provides the relative gene expression levels for each DEG across all samples. Trends of the DEGs are shown in light blue relative to the mean value. The right side of the panel provides a volcano plot summarising the differential gene expression analysis performed on RNA-seq data comparing TMZ-LC and CTRL. Red dots represent genes that were significantly upregulated (on the right side) or downregulated (on the left side). Dots in the volcano plot are colour-coded according to their statistical significance (FDR) and magnitude of expression change |log2FC|). The data were obtained from patient-derived GSC3 line, with three biological replicates performed for each of the conditions

**Fig. 5:**
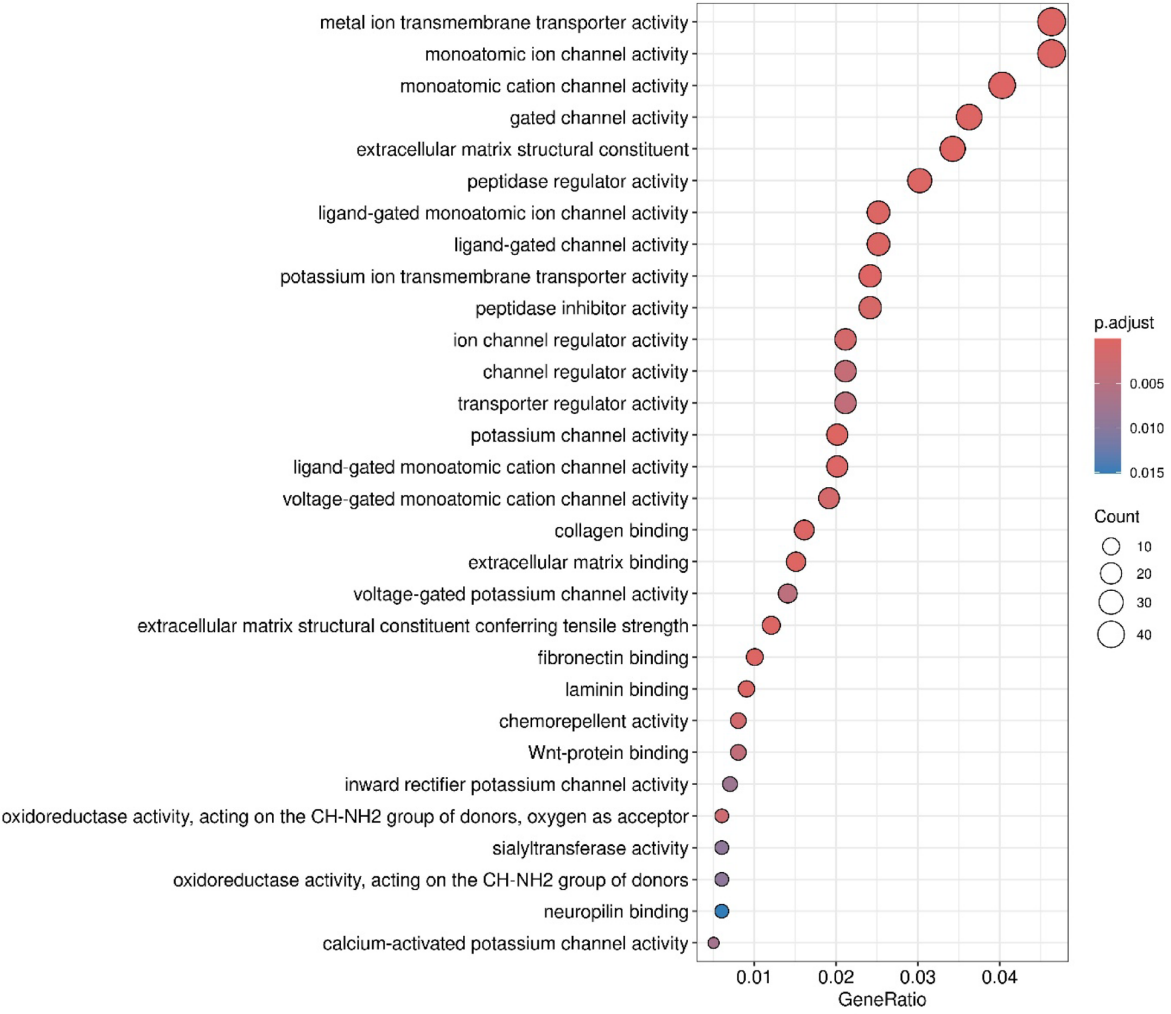
Gene Ontology (GO) molecular function (MF) enrichment analysis of DEGs between TMZ-LC and control GSC3. Results of the over-representation analysis (ORA) for Gene Ontology (GO) - Molecular Function (MF) of Gene Products were summarized and displayed as a dot plot. Analyses were performed using all genes with statistically significant differential expression levels identified by RNA-seq between TMZ-LC and CTRL samples (FDR < 0.05). The size of each dot in the plot corresponds to the number of differentially expressed gene products associated with each GO term, and the color of each dot represents the significance of enrichment represented by the BH-adjusted p-value

**Fig. 6:**
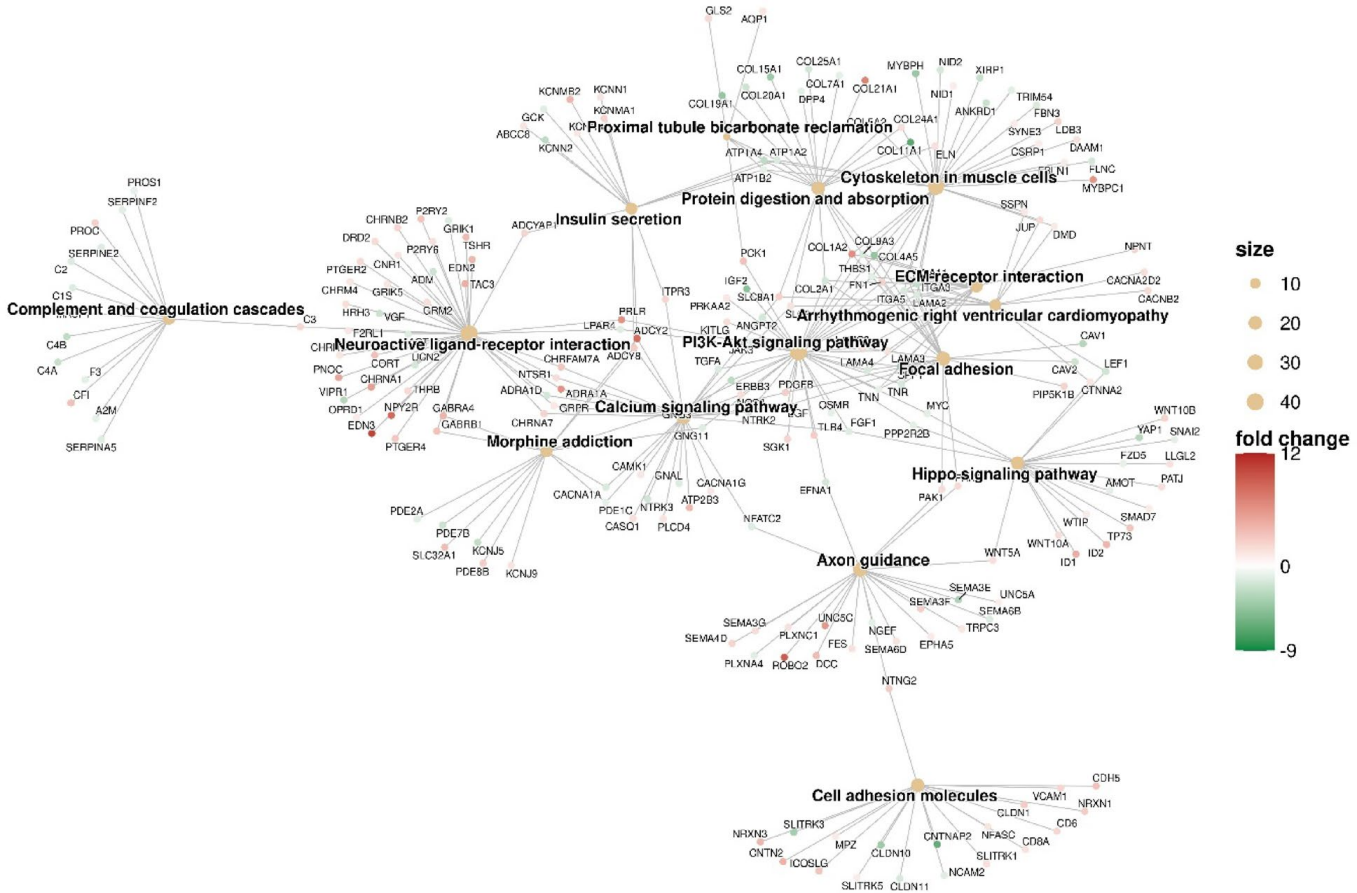
KEGG pathway enrichment network of DEGs between TMZ-LC vs. control GSC3. Network plot of over-representation analysis (ORA) using KEGG pathways (FDR < 0.05) between TMZ-LC and control GSC3 (CTRL). The size of the node illustrates the number of DEGs associated with that pathway. The colours of the nodes represent the fold change of DEGs in comparison to CTRL

Using cluster analysis, we identified differences between TMZ-Lc and Ctrl, as shown in a heatmap (Fig. 4, left panel), revealing distinct gene expression patterns unique to each cell type. The significantly upregulated and downregulated genes from the two groups were also visualized using a volcano plot analysis **(**Fig. 4, right panel). RNA sequencing revealed that out of 1,408 differentially expressed genes (based on a threshold of |log2 fold change| > 1) in TMZ-Lc-treated GSC3 line compared to Ctrl, 893 (63.4%) were upregulated and 515 (36.6%) were downregulated (**Fig. **S2). Figure 5 presents a Gene Ontology analysis within the molecular function (GO-MF) category, aimed at understanding the functions of the differentially expressed genes from RNA-Seq results in TMZ-Lc vs. Ctrl samples. The transcriptomic profile of GSC3 line exposed to a sub-lethal dose of TMZ (2.5 µM) showed a unique adaptive response, contrasting with the excitatory, synaptic-like phenotype seen after high-dose treatment (Figs. 5 and 6). GSC3 showed a significantly differential expression of stemness-related transcriptional regulators such as *ID1* and *ID2* (log2-fold change of + 4.54 and + 4.11, respectively); *WNT5A*, *WNT10A*, *WNT10B* (log2-fold change of + 1.72, + 1.91, + 2.44, respectively), and *TP73* (log2-fold change of + 3.19) belonging to the enriched Hippo signaling (Fig. 6). Interestingly, *YAP1* (log2-fold change of -3.01), usually considered a universal promoter of stemness, was downregulated, suggesting that a YAP1-independent mechanism might mediate CSC3 resistance to low-dose TMZ. A key feature of GBM is the significant reorganization of the extracellular matrix (ECM). The coordinated transcriptional alterations occurring to multiple components of a given pathway are captured via pathway enrichment analysis (rather than through uniform upregulation of individual genes). Therefore, YAP1 can experience downregulation while the Hippo signaling pathway still shows enrichment, especially in the scenario of how the differentially activated upstream Hippo kinases, transcriptional regulation factors, or YAP independent branches [35, 36]. TMZ-Lc-induced down-regulation of YAP1 in GSC3 might indicate that GSC have potentially entered a TGF-β/SMAD-mediated growth-suppressive or dormancy-associated transcriptional state, which is characteristic of a cell’s response to stress. Because of these findings, it is possible that by reducing YAP1 expression levels, cells may decrease their rate of proliferation and remain in an undifferentiated state that is characteristic of their microenvironment [37, 38]. 

In TMZ-treated cells, *FN1* (log2-fold change of + 2.38) and *COL21A1* (log2-fold change of + 7.02) were overexpressed. In contrast, *COL4A5* (log2-fold change of -4.42) and integrin alpha subunits 3 and 5 (*ITGA3/5*, with a log2-fold change of -2.52, -1.55, respectively) were downregulated in the enriched ECM-receptor interaction signaling. This indicates the presence of a fibronectin-rich, integrin-dependent stroma that supports survival through FAK/PI3K–Akt signaling. The increased expression of *PDGFB* (log2-fold change of + 2.64), *PCK1* (log2-fold change of + 3.48), *SGK1* (log2-fold change of + 2.33), and *COL1A2* (log2-fold change of + 6.41) in the PI3K–Akt pathway after low-dose TMZ further supports this idea, consistent with compensatory growth factor signaling and metabolic adaptation (Fig. 6). The neuroactive ligand-receptor interaction signaling showed an opposite trend compared to high-dose treatment, with upregulation of *NPY2R* (fold change of + 8.38), *EDN3* (log2-fold change of + 10.47). Similarly, *CDH5* (VE-cadherin, log2-fold change of + 3.44), and *VCAM1* (log2-fold change of + 2.33) were differentially upregulated in the cell-adhesion molecules signaling **(**Fig. 6). PI3K-Akt signaling, including *ERBB3* (log2-fold change of -2.74), *IGF2* (log2-fold change of -4.71), *VGF* (log2-fold change of -2.12), and *VIPR1* (log2-fold change of -3.02) was differentially downmodulated, indicating a shift toward a quiescent state and interaction with a potential perivascular niche. Meanwhile, the axon guidance signaling pathway tends to change roles from guiding axons to maintaining the niche, as shown by upregulation of Roundabout 2 (*ROBO2*, log2-fold change of + 9.2) and downregulation of semaphorins (*SEMAs*), especially *SEMA3E* and *SEMA6B* (with a log2-fold change of -3.25, -1.37, respectively) based on the KEGG analysis (Fig. 6). Notably, SEMA3E-plexinD1 signaling is essential for cancer cell survival and metastasis. Additionally, *SEMA3F* and *SEMA3G* (with log2-fold changes of + 2.69 and + 1.91, respectively), known as tumor suppressor SEMAs due to their roles in reducing cell adhesion, migration in vitro, and tumor blood vessel formation, increased significantly. The global profile may suggest that low-dose TMZ may foster the selection of dormant GSC3 associated with blood vessels, complicating treatment due to their niche interactions.

A variety of ATP-binding cassette (ABC) transporters have been reported to show increased expression in cancer stem cells, even though there is no single molecular GSC signature that applies to all cancers. The modification in the transport of endogenous metabolites and signaling molecules could be a factor in cancer development, and recent findings link ABC transporters to the stemness process [33]. Our analysis of transcriptomics in cells exposed to TMZ-Lc indicates a significant increase in the expression of some members of the family ABCA, including *ABCA4* (log2-fold change of + 2.71), *ABCA9* (log2-fold change of + 2.30), *ABCA7* (log2-fold change of + 1.14), *ABCA12* (log2-fold change of + 1.63), and *ABCC8* (log2-fold change of + 1.55). The ABCA family contributes to cholesterol homeostasis and the trafficking of membrane lipids; however, the upregulation of *ABCA4* has been shown to exhibit pro-oncogenic characteristics [39]. *ABCC8* is instead involved in the efflux of an essential mediator of inflammation, LTC4, which is derived from arachidonic acid and exerts a proinflammatory effect, facilitating the migration of immune cells to lymph nodes [40]. 

### High vs. Low TMZ: different paths to developing resistance

**Fig. 7:**
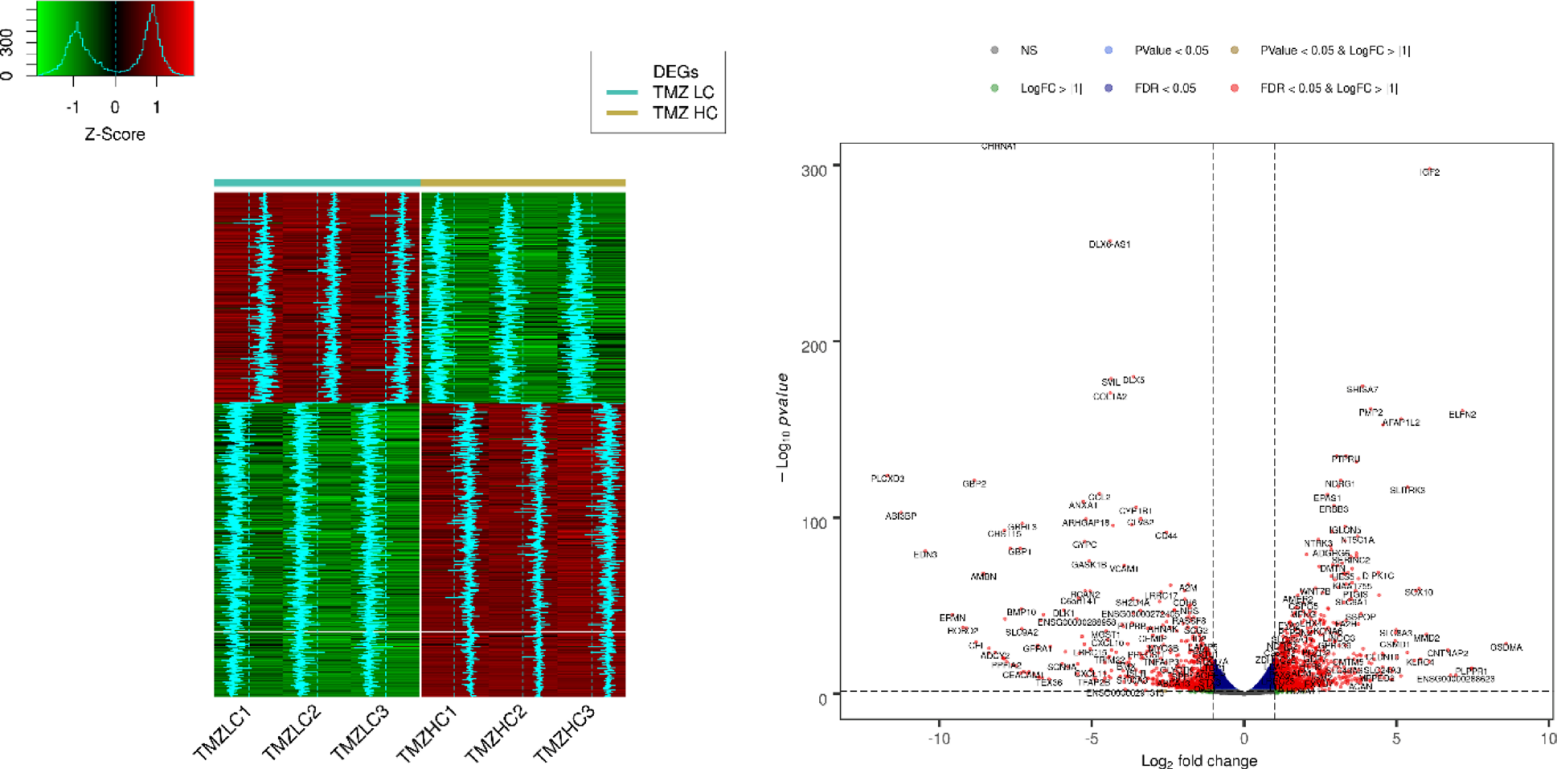
Comparative transcriptomic analysis between TMZ-HC vs. TMZ-LC. The left panel shows the heatmap patterns of differentially expressed genes (DEGs) between TMZ-HC and TMZ-LC conditions. Genes included meet the criteria of |log2FC| > 1 and FDR < 0.05. Heatmap values represent normalized gene expression levels scaled relative to the mean expression of each gene across all samples, with light blue indicating relative expression trends The right panel summarizes the Volcano plot of RNA-seq differential expression analysis comparing TMZ-HC vs. TMZ-LC conditions. Significantly up- and down-regulated genes are highlighted in red. Points are color-coded according to combinations of statistical significance (FDR) and magnitude of expression change (|log2FC|), as indicated

**Fig. 8:**
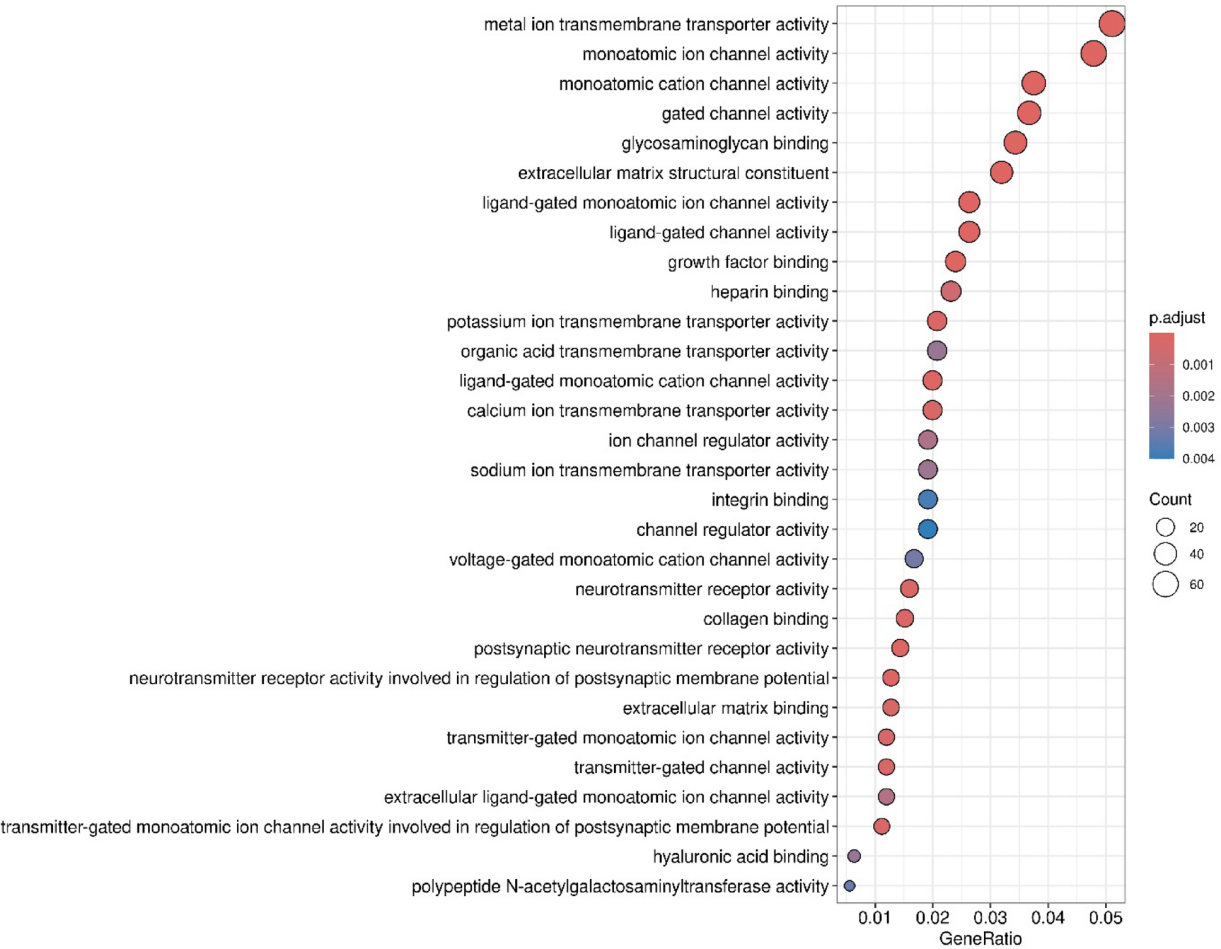
Gene Ontology (GO) molecular function (MF) enrichment analysis of differentially expressed genes between TMZ-HC vs. TMZ-LC. Dot plots illustrate the results of an Over Representation Analysis (ORA) utilizing Gene Ontology (GO) in the Molecular Function category, which represents the biochemical functions associated with the gene product. The analysis was conducted on differentially expressed genes from the RNA-seq between TMZ-HC and TMZ-LC based on their adjusted p-values < 0.05. The size of the dots in the plot reflects the number of DEGs associated with that GO term, while the color indicates the P-adjusted values (BH)

**Fig. 9:**
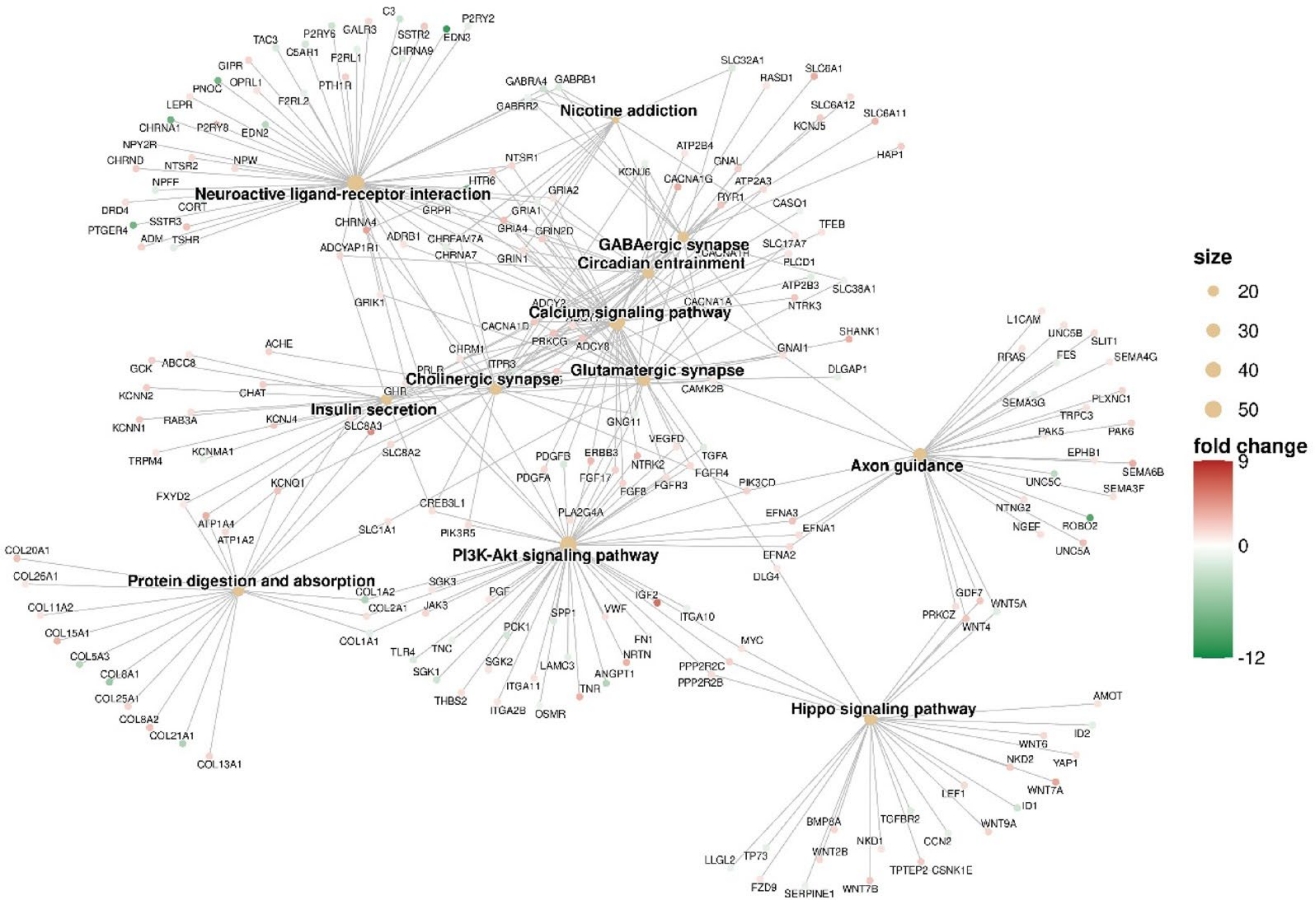
KEGG pathway enrichment network of DEGs between TMZ-HC vs. TMZ-LC. The functional profile illustrates the relationship between DEGs and KEGG pathways across groups. Sizes indicate the number of differentially expressed genes belonging to the enriched KEGG pathways. Fold change represents the difference in fold change between TMZ-HC and TMZ-LC

By employing cluster analysis, we observed variations between TMZ-hc and TMZ-Lc cells, as shown in the heatmap **(**Fig. 7, left panel), which revealed unique gene expression profiles for each cell type. The significantly upregulated and downregulated genes from the two distinct populations were also illustrated through a volcano plot analysis **(**Fig. 7, right panel). We identified 1,944 genes showing differential expression (meeting the criterion of |log2 fold change| > 1) in GSC3 line exposed to high doses of TMZ (12.5 µM) compared to those treated with a lower dose of TMZ (2.5 µM). Notably, 1,134 (58.3%) of these genes were upregulated, while 810 (41.7%) were downregulated (**Fig. **S3). As shown in Fig. 8, a GO analysis focusing on the molecular function (GO-MF) category was performed to clarify the roles of the differentially expressed genes obtained from RNA-Seq data in TMZ-hc vs. TMZ-Lc samples. The analysis of high- and low-dose TMZ treatment revealed two distinct adaptive responses in GSC3, confirming a specialized excitatory synaptic-like phenotype **(**Fig. 9**)**, characterized by hyperactivation of Ca+-regulated adenylate cyclase family members, including *ADCY1*, *ADCY5*, *ADCY8* (with a log2-fold change of + 1.35, + 1.96, + 2.3, respectively) and neuroactive ligand-receptor genes (*GRIN1*, *GRID2*, *GRIA2*, *GRIA4*, *SSTR2*, *SSTR3*, *CHRM1* (with a log2-fold change of + 1.44, + 2.06, + 1.13, + 2.73, + 2.31, + 2.60, and + 1.15, respectively). Based on current data, while the majority of ADCY family members were found to be significantly increased in expression, this does not provide direct evidence that ADCY activity is responsible in any way for TMZ resistance. Instead, we see the upregulation of the ADCY’s to be part of a larger network of adaptive signaling pathways stimulated by exposure to genotoxic stress, leading to increased survival through cAMP-dependent mechanisms. Thus, we define ADCY upregulation to be an adaptive process rather than mechanistic evidence of support for TMZ resistance.

Additionally, the scaffold protein SH3 and multiple ankyrin repeat domain protein 1 (*SHANK1*, log2-fold change of + 3.45) were significantly upregulated in the glutamatergic signaling pathway of TMZ-hc GSC3. Several voltage-gated Ca2^+^ channel subunits (*CACNA1A/G/H/D*, with a log2-fold change of + 2.03, + 3.54, + 1.38, + 2.41, respectively) were upregulated in TMZ-hc, as indicated in the GO and KEGG pathways analyses **(**Fig. 9). Moreover, GSC3 line exposed to high-dose TMZ showed higher levels of *SLC17A7* (*VGLUT1*, log2-fold change of + 1.06) compared to those exposed to low-dose TMZ, suggesting enhanced vesicular glutamate processing and potential excitatory autocrine pathways.

In contrast, *GABRA4*, *GABRB1*, and *GABRR2* (with a log2-fold change of -2.87, -1.92, and − 1.02, respectively), which encode the α2, β3, and Rho-2 subunits of the GABA-A receptor, were downregulated, indicating decreased inhibitory signaling and a shift toward hyperexcitability. Overall, our findings suggest that resistant GSC3 alter their neurotransmitter receptors and ion channel composition at the transcriptional level to increase excitatory and calcium-mediated signaling while reducing inhibitory GABAergic inputs.

We also observed differential activation of neurotrophic tyrosine kinase receptors *NTRK2* and *NTRK3* (with a log2-fold change of + 3.09, + 2.43, respectively), both crucial for neuronal survival, plasticity, and excitatory synapse formation as indicated in the PI3K-Akt and glutamatergic synapse signaling pathways (Figs. 8 and 9). These changes support the hypothesis of a neuroactive, synaptic-like program and reveal how TMZ-hc cells exploit brain neurotransmission pathways to maintain viability and function. High doses of TMZ will preferentially enhance the expression of synaptic- and neuroactive ligand–receptor-related genes. Although not indicative of neuronal activity or function, this enrichment pattern is suggestive of genomic responses to cytotoxic stress.

Suppressor of cytokine signaling type 2 (*SOCS2*), a negative regulator of JAK/Stat signaling, was downregulated (log2-fold change of -2.37).

**Fig. 10:**
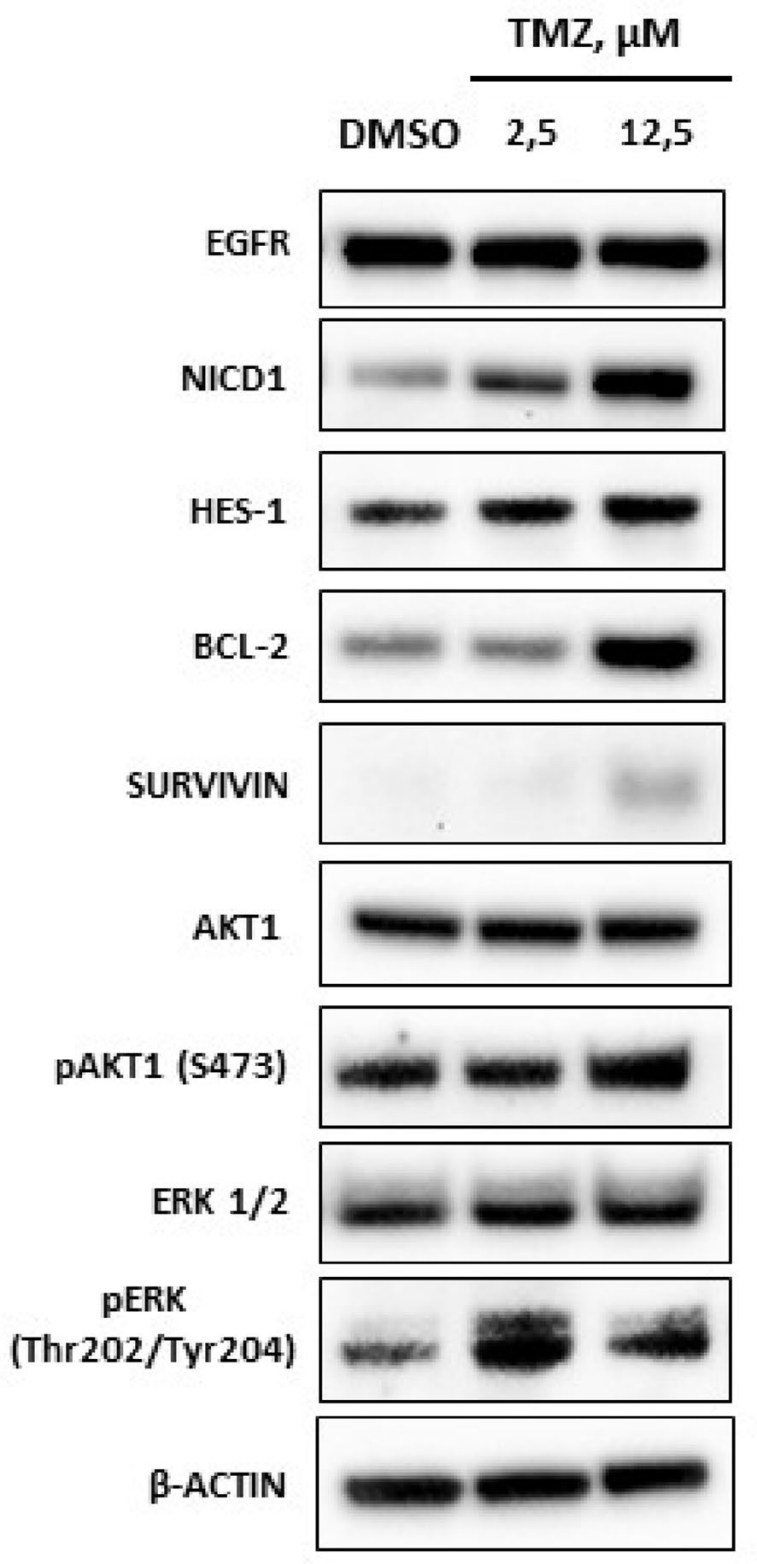
Western blot analysis of signaling pathway activation in TMZ-treated vs. control GSC3. A protein analysis was performed to determine the levels of NICD1, Bcl-2, and Survivin in GCS3 treated with 2.5 µM and 12.5 µM TMZ, respectively. In TMZ-HC conditions, there is a significant increase in the levels of all three proteins compared to TMZ-LC and CTRL. Total levels of EGFR, Akt1, and Erk1/2 were not significantly different between the CTRL and high or low-dose of TMZ-treated GSC3, confirming the results obtained from transcriptomics analysis. On the contrary, phosphorylated p-Akt1 and p-Erk1/2 were markedly elevated in both TMZ treatments compared to CTRL. The images provided are representative of two independent biological experiments performed in duplicate

Activation of the Notch pathway by *DLL1* (log2-fold change of + 1.23), along with target effectors *HES5* and *HES7* (log2-fold change of + 3.25 and + 2.85, respectively) and the modulator *DNER* (log2-fold change of + 1.51), most likely contribute, through both paracrine and autocrine mechanisms, to the self-renewal of TMZ-hc compared to TMZ-Lc. These changes were supported by Western blot analyses (Fig. 10), which show upregulation of active Notch1 (NICD1) and accumulation of anti-apoptotic proteins Bcl2 and Survivin, indicating that the GSC3 line was in a state of reactivation and adaptive survival (Fig. 10).

In the transcriptomic analysis, the accumulation of the cleaved Notch1 intracellular domain (NICD1) and the enrichment of Notch-associated genes suggest that Notch signaling may be implicated in GSC3 line resistance to TMZ-hc treatment. However, to fully support the conclusion of activation of canonical Notch signaling, independent verification at either the mRNA or protein level for transcriptional activation of downstream targets is necessary; therefore, Notch signaling has been interpreted as a potential involvement/association with resistance to TMZ treatment rather than definitive proof. Future experiments must include targeted cloning and validation of canonical Notch target genes, as well as the use of reporter-based assays to quantify Notch transcriptional activity.

The *HOX* (homeobox) gene family has garnered considerable attention due to its involvement in cellular differentiation, development, and cancer biology. They rigorously control the processes of tumor initiation and growth, invasion and metastasis, angiogenesis, resistance to anti-cancer therapies, and the origins of stem cells. In this study, we observed elevated levels of *HOXA5*,* HOXA11*,* HOXA13*, and *HOXB4* (log2-fold changes of + 1.28, + 1.95, + 1.63, and + 1.18, respectively) in TMZ-hc compared to low-dose treatment, highlighting their significance in tumor progression and resistance to therapy.

Additionally, our findings reveal the upregulation of components involved in Wnt signaling (Fig. 9), specifically *WNT7A* and *WNT7B* (log2-fold changes of + 3.74 and + 2.33, respectively), along with downstream factors such as *HOXA13* (log2-fold change of + 1.63) and *LEF1* (log2-fold change of + 1.51). Collectively, these factors are crucial for maintaining stem cells and facilitating the epithelial-to-mesenchymal transition (EMT) process, which enables cell migration and invasion across various cancer types, including GBM [27]. It is essential to note the discovery made by Griveau et al. that reveals a close correlation between the expression of *WNT7A* and *WNT7B* with oligodendrocyte precursor cell (OPCs) markers in high-grade and proneural GBM [28]. This finding is notably compelling, particularly because Olig2^+^ GBM advance at a faster rate than Olig2^−^ tumors. We might speculate that TMZ-hc GSC3 line could present a more aggressive phenotype when faced with genotoxic stress compared to TMZ-Lc.

An increased expression of *FGFR3* and *FGFR4* (with a log2-fold change of + 1.44, and + 1.47, respectively) was identified, linked to the PI3K-Akt signaling pathway (Fig. 10), which aligns with the more aggressive features of TMZ-hc GSC3 line. In fact, fibroblast growth factor receptors (FGFRs) represent promising targets for cancer treatment, particularly given that their overexpression and hyperactivation have been detected in an exceptionally aggressive subset of GBM.

Similarly, ephrin receptors, part of the RTK family, are involved in various cancer processes. Overexpression of both class A and B ephrin receptors is associated with more aggressive and metastatic tumors, consistent with the roles of ephrin in regulating axonal guidance, cell migration, and angiogenesis. Consequently, the upregulation of *EPHA10* and *EPHB1* (with a log2-fold change of + 2.34, + 1.18, respectively) observed in TMZ-hc GSC3 line supports their potential role in promoting GSCs growth. This transcriptional program correlates strongly with elevated *YAP1* expression (log2-fold change of + 1.24) in TMZ-hc GSC3 line, indicating that the Hippo signaling plays a key role in reactivating these cells.

Moreover, our findings suggest that the increased expression of Netrin-1 receptors, particularly *UNC5A* and *UNC5B* (with a log2-fold change of + 2.76 and + 1.29, respectively), is likely linked to heightened invasiveness and self-renewal capabilities of GSC3 in TMZ-hc. With changes in axonal guidance signals and dependence-receptor signaling specific to the dosage of TMZ, the decreased expression of the axonal guidance receptor *ROBO2* (log2-fold change of -9.22) also suggests a more proliferative phenotype in TMZ-hc as compared to low-dose treatment (Fig. 9).

Based on our findings, we propose that at least two different but complementary mechanisms contribute to TMZ resistance in GSC3. An excitatory reprogramming triggered by high doses enhances survival signaling while impairing DNA repair. On the other hand, preserving stemness and microenvironmental signals, hypothetically seems to be crucial during lower-dose treatment, as it encourages cell quiescence. While TMZ resistance involves both intrinsic signaling changes and external niche factors, this paradox emphasizing the complexities in formulating successful therapies.

### Oncogenic signaling pathways modulation in TMZ-treated GSC3 line

Western blot analysis revealed that various pro-survival pathways were activated in GSC3 line treated with TMZ compared to control cells **(**Fig. 10**)**. There is strong evidence from transcriptomic signatures and protein level observations that show signaling pathways associated with cell survival (including Notch-related programs) to be engaged in TMZ-hc cells. These findings provide additional support for their involvement but highlight the importance of performing targeted functional validation for confirming canonical activation of these pathways.

Notably, we observed a significant increase in the cleaved Notch1 intracellular domain (NICD1) and its transcriptional target HES1 **(Table **S4**)**. The Notch1 pathway plays a crucial role in the self-renewal and differentiation of neural and embryonic stem cells, as well as in tumorigenesis. The anti-apoptotic proteins Bcl-2 and Survivin also exhibited a significant upregulation in TMZ-hc compared to TMZ-Lc and control cells **(Table **S4**)**, consistent with their function in conferring resistance to anticancer therapies [14, 41–43]. Furthermore, the activation of Akt1 (pAkt1), known for its role in promoting cellular growth and increasing resistance to apoptosis, was markedly elevated in the TMZ-hc condition compared to the control cells. In contrast, pErk exhibited significant modulation in both TMZ conditions. **(Table **S4**).** Although the increase in NICD1 may indicate involvement of the NOTCH signaling pathway, because downstream transcription factor molecules (HES5, HES7) have not been independently verified, conclusions regarding complete activation of the canonical NOTCH pathway are weak. Previous studies have shown that NICD1 can accumulate without significant activation of transcription from the HES family, depending on environmental factors (such as the surrounding chromatin), the availability of cofactors, and other stressors [44, 45]. We cannot draw firm conclusions about the transcriptomic response to the Notch pathway due to the absence of supplementary data supporting this response. The primary conclusions drawn from this research were based on transcriptomic signal correlation instead of direct functional genetic validation. Therefore, the mechanistic interpretations presented may only be regarded as models supported by observation, rather than definitive causal relationships.

Our transcriptomics research reveals that TMZ resistance in cancer stem cells is not a singular mechanistic occurrence, but rather a multifaceted adaptive process involving synergistic signaling, transcriptional, and microenvironmental pathways that enhance their ability to maintain stem cell-like characteristics, survive, and evade apoptosis. The analyses reveal the acquisition of a neuronal-like identity when cells are exposed to high doses of TMZ, accompanied by the emergence of a quiescent, niche-dependent phenotype under low-dose conditions. The neuronal and synaptic features illustrated in this study are based on transcriptomic enrichment analyses. Consequently, it is important to note that functional validations, including electrophysiological recordings of color changes in response to stimuli, calcium imaging, and neurotransmitter release assays, were excluded from this study because of existing constraints.

The findings of this research indicate a clear relationship between the amounts of TMZ exposure and the GSC3 that adjust in response, but the key molecular mechanisms triggered by this adaptation remain uncertain at this time. Different but complementary mechanisms demonstrate the adaptability of GSC3 and their ability to utilize both internal signaling pathways and external environmental stimuli. Notch1 and anti-apoptotic signaling (Fig. 10) activation reported in TMZ-hc cultures corresponds to their recognized functions in cancer stem cell biology, which involve maintaining an undifferentiated stem-like state and, consequently, inhibiting differentiation pathways that might make them more vulnerable to DNA-damaging treatments [10, 36]. Furthermore, together with the downregulation of SOCS2/3, it positions resistant GSC3 line at the center of unregulated STAT3 activity and reduced DNA-damage responses, thereby intensifying resistance [12, 46]. Although transcript levels remain constant, the increased amount of survivin at the protein level suggests the existence of post-transcriptional mechanisms that stabilize it. Survivin is associated with the inhibition of PARP cleavage and apoptosis induced by DNA damage in GBM, and its overexpression in this context strongly reinforces its function as a key mediator of resistance [14]. 

In addition to conventional survival pathways, we observed a pronounced activation of a synaptic-like neuroactive program (Figs. 3, 8 and 9) in TMZ-hc cells. This is indicated by a significant increase in the levels of specific neurotransmitter receptors, such as *GRIN1*, *GRIA4*, *DRD2*, and *HTR6*, ion channels such as *CACNA1G/D/H* and *KCNB1*, and signaling nodes, such as the *ADCY* family, and its upstream and downstream regulators, recognized as potential targets for novel anticancer treatments [47]. These changes suggest that resistant cancer stem cells may utilize both autocrine and paracrine signaling pathways, activating Ca²⁺ and cAMP pathways to enhance pro-survival transcription factors, such as CREB [48, 49]. Although the increase in the expression of *ADCY* indicates a higher ability for cAMP signaling, the current findings do not show a direct cause-and-effect relationship between the activation of ADCY and being supportive of GSC3 to survive in the presence of TMZ. The framework discussed in this text is a preliminary means of grasping how we can consolidate all the information we have gathered about the different types of molecular changes that occur following exposure to an environmental stimulus. It ought to be viewed as a potential for generating new hypotheses, though it has not yet been tested for reliability. Downregulation of inhibitory GABA-A receptor subunits (*GABRA4*, *GABRB1*, and *GABRR2*) worsens this imbalance, making it even more hyperexcitable. Conversely, TMZ-Lc treatment enhances GABAergic synaptic transmission by regulating GABA receptor subunits *GABRA4*, *GABRB1*, and *GABRR2*. These neuronal alterations may facilitate the integration of GSC3 line into neural circuits, thereby enhancing their survival and invasiveness in response to chemotherapy stress [16]. To determine whether decreased transcriptional activity for GABA receptor subunits results in lowered levels of inhibition and plays a part in the development of resistance against TMZ, future research studies need to perform validation via protein expression and through functional assays.

*MLH1* gene inhibition in resistant GSC3 represents a conventional mechanism of TMZ resistance, since the lack of mismatch repair machinery increases DNA damage, but does not diminish apoptosis following the formation of O6-methylguanine adducts [50]. *MLH1* suppression, therefore, induces a hypermutator phenotype, which promotes clonal diversification and contributes to the maintenance of tumor heterogeneity and aggressive recurrence [51, 52]. 

Exposure of cells to low doses of TMZ triggered an alternative resistance pathway, characterized by cellular quiescence, increased stemness factors, and niche engagement. Increased levels of *ID1*,* ID2*,* WNT5A*, and *WNT10A/B* suggest a transcriptional mechanism that supports self-renewal [6, 8]. Even though Hippo pathway enrichment is usually considered to represent YAP or TAZ activation under conditions of proliferation, the growing body of evidence indicates that Hippo signaling can also positively influence cellular quiescence (growth arrest), differentiation, and stress adaptation independently of the action of YAP. Therefore, the fact that YAP1 is downregulated by low concentrations of TMZ does not contradict the data indicating that Hippo pathway enrichment and transcriptional reprogramming of YAP1 towards quiescent and microenvironment-dependent behaviors occur as a result of low concentrations of TMZ [53, 54]. 

Several components of the Hippo signaling pathway, such as MST1/2, LATS1/2, and TEAD-associated cofactors, have been identified to regulate transcriptional programs that are independent of YAP1. These components likely influence the adaptive resistance that arises from low-dose exposure to temozolomide, rather than engaging YAP1 activation. In fact, the components MST1/2, LATS1/2, and TEAD cofactors are thought to regulate transcriptional responses to metabolic stress or DNA damage [55, 56]. 

IDs are associated with purine metabolism and the development of chemotherapy resistance in ID1-high GBM cells [57]. Recently, ID1 has been recognized as a key regulator of tumor vascular mimicry (VM), a process that allows tumors to create vessel-like structures without relying on tumor angiogenesis, thereby facilitating cancer progression [27]. Additional evidence for ID1’s function in VM formation is its regulation of PDGFB, a known regulator of VEGF expression in cancer and in cellular resistance, which is consistent with our data from TMZ-Lc GSC3 line. Activation of the WNT pathway protects tumor stem cells from genotoxic stress [58]. Aberrant activation of canonical Wnt/β-catenin signaling is responsible for many pathological processes, such as cancer, inflammatory and immune diseases, and metabolic disorders [27]. Findings obtained from the Cancer Genome Atlas (TCGA) and the Chinese Glioma Genome Atlas (CGGA) indicate that *WNT5A* expression is significantly increased in glioma, which relates to a shorter overall survival (OS); on the other hand, *WNT10B* serves as a glioma suppressor, with lower levels of expression in glioma being associated with enhanced OS outcomes for patients [59]. Concurrently, the use of PORCN inhibitors has been shown in preclinical studies to increase the sensitivity of gliomas to TMZ, offering a promising avenue for targeted treatment of Wnt-driven cancers [60]. 

Another noteworthy alteration was the change in the shape of the extracellular matrix (ECM). Levels of FN1 and *COL1A2* increased, while integrins, especially *ITGA3/5*, decreased. This suggests that the ECM is undergoing restructuring into a network rich in fibronectin, integrin, and FAK–PI3K–Akt signaling pathways that promote survival. Previous studies have shown that α5β1 integrin-mediated FAK signaling inhibits p53 and contributes to chemoresistance [61]. In this context, increased *PDGFB* expression promotes compensatory receptor tyrosine kinase (RTK) signaling, which is consistent with the established role of *PDGFR* in regulating GSCs proliferation and their resistance to TMZ [62]. 

Metabolic adaptation, demonstrated by *PCK1* gene overexpression, has emerged as an auxiliary mechanism and, together with *ID1/ID2*, can enhance one-carbon metabolism and purine synthesis. Consistently, recent studies have confirmed that resistant GSCs rely on purine biosynthesis to conserve nucleotide pools, and blocking this pathway can restore TMZ sensitivity [63]. Vascular mimicry markers, including *CDH5* (VE-cadherin) and *VCAM1*, may support the preservation of GSC3 in a dormant state within their niche, enhancing their interaction with blood vessels and offering protection against therapeutic measures [64]. The strong upregulation of *EDN3* and neuronal adhesion molecules, such as *NRXN1/3* (known as Neurexins1/3) and *CNTN2* (known as Contactin-2), suggests that TMZ-Lc cells may leverage not only neuronal but also vascular signals for survival, consistent with findings that GSC3 can assimilate into neurovascular niches and use endothelin signaling for sustenance [7, 65]. 

An alternative strategy used by GSC3 to adapt to TMZ-Lc involves regulating semaphorins (*SEMAs*), which were initially identified as guidance factors aiding axon pathfinding during nervous system development. However, they have since been recognized for their roles in controlling angiogenesis, lymphomagenesis, and immune responses, as well as their influence on tumor growth and invasion. Importantly, we have demonstrated that the upregulation of SEMA3F and SEMA3G in tumor cells, which are known as tumor suppressor genes due to their inhibitory effects on angiogenesis, proliferation, adhesion, migration, and invasion in various cancers, including glioma, is highly significant. Specifically, research by Futamura et al. demonstrated that the tumor suppressor protein p53 reduces tumor angiogenesis and cell proliferation via the SEMA3F-Neuropilin-2 (NRP2) pathway [66]. In melanoma, *SEMA3F* acts as a chemorepulsive factor for endothelial cells, thereby decreasing lymphatic vessel formation and lymphatic metastasis in vivo [67]. Conversely, several SEMAs, including SEMA4D, SEMA5A, SEMA6A, and SEMA7A, exhibit pro-angiogenic properties. Our results reveal that *SEMA4D* is differentially upregulated in TMZ-Lc GSC3, though is mainly produced by tumor-associated macrophages (TAMs) within the tumor stroma [68]. *SEMA4D* can be processed and released as a soluble form by activated MMP-1, which induces chemotaxis in endothelial cells and promotes blood vessel formation in vivo.

In summary, our research highlights two distinct yet complementary mechanisms through which GSC3 line adapt to TMZ: a high-dose TMZ-responsive mechanism involving activation of excitatory synaptic signaling, maintenance of stemness, and inhibition of DNA repair; and a low-dose drug-responsive mechanism characterized by entry into a quiescent state **(**Fig. 6), preservation of stemness, extracellular matrix remodeling, and targeted interactions within a neurovascular niche. Both pathways present significant treatment challenges due to the interplay between innate cellular resistance and microenvironmental support.

From a translational perspective, our analysis reveals several potential intervention options. The Notch and Stat3 pathways remain serious candidates for pharmacological suppression, with γ-secretase inhibitors and Stat3 blockers currently in clinical development [69, 70] Survivin is another interesting potential target for anticancer therapy. In this context, the novel small-molecule survivin inhibitor YM155 has demonstrated antiproliferative activity in preclinical models of GBM [71]. Finally, targeting ECM-mediated survival via integrin or FAK inhibitors, or using PDGFB-induced RTK signaling with PDGFR-targeted therapies, may improve the efficacy of TMZ in resistance settings [34, 35, 49, 58]. 

Furthermore, PORCN inhibitors that decrease Wnt production and purine metabolism inhibitors represent potential therapeutic options to counteract stemness and metabolic shifts caused by low doses of TMZ [7, 55]. Finally, disrupting tumor-neuron connections, either by inhibiting *CNTN2* production or targeting *EDN3* signaling, may provide an innovative strategy for dismantling neuroactive resistance mechanisms, leading to cell apoptosis and functional impairment of tumor sphere formation and cell migration.

However, our study still has some limitations. Bulk RNA sequencing does not fully capture the variability of resistance states; therefore, single-cell or spatial transcriptomics are essential to clarify the distribution of these programs in the perivascular and neuronal-adjacent environments. Moreover, it is crucial to verify the causal role of potential factors, such as ID1, PDGFB, FN1, and EDN3, in resistance through loss-of-function and gain-of-function experiments. Finally, effective translation requires accurate patient classification, as previous GBM studies targeting integrins, PDGFR, or other pathways have been unsuccessful when applied broadly without molecular selection [48, 49]. 

The lack of functional synaptic or electrophysiological assessments restricts the ability to validate the synaptic-like transcriptional program determined by transcriptomic analysis. Although YAP/TAZ’s subcellular localization and function have been assessed at the transcriptomic level, functional assessments of the Hippo pathway have not yet been conducted. Thus, it is not currently known if the mechanistic roles observed in the Hippo pathway are due to YAP-dependent or independent mechanisms. It is also necessary to conduct future studies using both transporter-selective inhibition and genetic modulator manipulation to find out whether or not ABCG4 contributes to drug transport, lipid metabolism, or stress resistance in glioblastoma stem cells. That wasn’t exactly what we wanted to achieve with this experiment. Specifically, while we have shown previously that canonical Notch signaling includes HES5 and HES7 as direct downstream targets, these genes were not validated as independent qPCR or immunoblot targets. Hence, in order to determine the degree of functional significance of Notch pathway involvement in TMZ-resistant GSC3, it will be important to measure its activity directly in future investigations.

One limitation of this study is the reliance on merely two experiments (*n* = 2) performed in duplicate for the Western blot analysis. Consequently, the statistical power is constrained, but we have documented the consistent protein changes that support our comprehensive transcriptomic analysis through densitometry analysis. Future research will require the inclusion of more replicates and quantitative validation.

Another main limitation of this research is that there are no experimental tests to determine whether or not individual signaling pathways or transcription factors can cause the development of resistance to TMZ. Therefore, it is not possible to definitively say that these signaling pathways or transcription factors are causing resistance to TMZ. Notwithstanding the previously mentioned limitations, the similarities in the observed dose-responsive transcription patterns confirm the existence of an adaptive state that is robust, and is a direct result of the consensus across the multiple analyses.

By integrating the dose-dependent transcriptional programs identified in this research with our prior observations (manuscript in preparation), which show overexpression of dysadherin (*FXYD5*) and downregulation of *ROBO2*, a receptor in the SLIT–ROBO pathway, we propose a cohesive mechanism by which glioma stem cells gain resistance to TMZ. Indeed, FXYD5 plays a role in modulating Na⁺/K⁺-ATPase and facilitates the activation of NF-κB and Akt, thereby enhancing survival and invasion in stressful environments [72, 73]. 

Surprisingly, we observed a downregulation of *FXYD5* and *FXYD7* in TMZ-Lc cells compared to TMZ-hc. In contrast, the overexpression of Roundabout 2 (*ROBO2*) in TMZ-Lc inhibits migration and proliferation, while suppression of *ROBO2* in TMZ-hc negates dependence-receptor modulation of apoptosis, increases invasive plasticity, and fosters neuron–tumor synaptic integration [16, 74–77]. Concurrently, a reduction in actin-binding protein dematin (*DMTN*) encourages GBM proliferation and invasion by affecting cell cycle regulation and actin remodeling [78]. This model predicts how low-dose TMZ induces a chemotherapy-tolerant state, similar to a dormant state characterized by the downregulation of *FXYD5/7*, the upregulation of *ROBO2*, and a decrease in *DMTN*. In contrast, high-dose TMZ more strongly activates excitatory synapses through the activation of *NTRK2* and *EPHA10* pro-survival signaling, ultimately increasing resistance. This suggests that effective treatment strategies must target ion-pump-related signaling (FXYD5 axis), dependence-receptor integrity (ROBO/SLIT), and cytoskeletal dynamics simultaneously, while inhibiting the canonical PI3K/Akt and Wnt pathways, to prevent persistent resistance to TMZ [16, 78, 79]. 

TMZ therapy in our dataset suggests the activation of a neuronal-associated/neuroactive gene expression enrichment in GSC3 in a dose-dependent manner, as evidenced by the significant increase in neuronal markers such as *NTRK2*, *NTRK3*, and *TUBB4A* in TMZ-hc. This may indicate a genuine differentiation accompanied by loss of self-renewal capacity in a minority of cells, or the selection or priming of a neuronal-like cell that retains resistance characteristics [35, 80, 81]. 

BDNF/TrkB signaling activates the pro-survival PI3K/Akt and MAPK pathways in neural and glioma cells and is correlated with growth, resistance to treatment, and invasive behavior in glioma models [82, 83]. This suggests that the induction of NTRK2 creates a pro-survival signaling pathway, allowing neuronal-activated cells to survive after high-dose TMZ treatment. Indeed, pharmacological or genetic inhibition of TrkB has demonstrated the ability to sensitize glioma cells in preclinical studies, thus supporting the idea that TrkB plays a functional role in TMZ tolerance under TMZ-hc conditions [83, 84]. 

Our data show that TMZ-hc administration does not trigger a universal mechanism for multidrug resistance; instead, it may alter the utilization of transporters. The increased levels of *ABCC8* and *ABCG4* imply that channel regulation and cholesterol/lipid management serve as adaptive strategies for survival. In contrast, reduced expressions of *ABCC3* and *ABCA13* indicate that specific efflux pathways are suppressed, which paradoxically increases the susceptibility of GSCs to TMZ-induced cytotoxicity. This observation is consistent with previous research demonstrating that GBM cells utilize ABC transporters in diverse ways to evade chemotherapy, and that both environmental factors and dosage influence the expression levels of these transporters [85, 86]. The lack of *ABCC3* and *ABCA13* may explain the increased vulnerability of GSC3 line to elevated doses of TMZ, as these transporters generally promote drug efflux and metabolic detoxification. This finding is consistent with reports for other malignancies, where reduced MRP3 expression correlates with enhanced sensitivity to DNA-damaging agents [87, 88]. The data that presently exists does not enable us to define how ABCG4 contributes to the efflux of temozolomide or the ability of GSC3 line to develop resistant to this drug; however, ABCG4 is included within the group of ABC transporters upregulated in TMZ-hc GSC3 because it has been identified as a marker for altered transporter activity or metabolic adaptation to TMZ-related pressures rather than directly associated with the efflux mechanism for temozolomide.

### Therapeutic considerations

Our findings suggest several therapeutic options. First, targeting survivin seems to be the most urgent strategy, as its inhibition reinitiates apoptosis in resistant glioma cells [12]. Second, disrupting neuroactive signaling through the use of DRD2 antagonists (e.g., ONC201) or techniques that prevent the formation of glutamatergic synapses could reduce the excitatory pathways characteristic of cells still surviving after exposure to high doses of TMZ. Third, restoring the activity of dependency receptors (ROBO2/UNC5C) can restart ligand-independent apoptosis and limit invasion [76]. Finally, blocking RTK/PI3K/FAK is an effective way to counteract the low-dose, niche-dependent adaptation program. Blocking the Wnt pathway (e.g., using PORCN inhibitors) directly affects stemness and adaptive survival mechanisms activated by *Wnt7A* and *Wnt7B*. These strategies suggest that combination therapies targeting synaptic, survival, and microenvironmental pathways simultaneously hold the greatest potential to overcome TMZ resistance in GBM [77]. 

Taken together, this work provides a framework for understanding the role and influence of chemotherapeutic agents on the adaptation of transcriptional states in GSC3 line as well as the ability to generate hypotheses for the development of future genetic and functional studies.

## Conclusions

The results of this research underscore the need for further substantial future investigations. First, the causal link between the activation of the identified pathways and the resistant phenotype must be clearly confirmed through rigorous functional genetics. Techniques such as siRNA, shRNA, or CRISPR-Cas9-mediated knockdown of potential driver cancer genes, including *ID1*,* BIRC5*,* NTRK2*,* ERBB3*,* EPHRA10*,* EDN3*,* WNTs*, or *DRD2*, in resistant GBM stem-like cells, will help determine whether blocking these pathways can restore cellular sensitivity to TMZ. Furthermore, it is essential to conduct preclinical therapeutic evaluations of these pathways to determine their potential as drug targets.

Patient-derived orthotopic xenograft models would provide a physiologically relevant platform for testing combination strategies that include TMZ with inhibitors such as γ-secretase inhibitors (targeting Notch1), Stattic or WP1066 (targeting Stat3), the survivin inhibitor YM155, or ONC201 (a DRD2 antagonist with known anti-GBM activity). Success in these models would establish a translational foundation for rational combination therapies aimed at eliminating resistant CSC subpopulations. The cellular heterogeneity that leads to TMZ resistance also requires further investigation. Our transcriptome analysis revealed distinct activation of multiple pathways. Nonetheless, single-cell RNA sequencing (scRNA-seq) of TMZ-treated and untreated GSCs, in conjunction with corresponding patient tumors, is essential to determine whether these programs coexist within the same subpopulation or represent separate, mutually exclusive resistance mechanisms. This distinction is clinically crucial, as it could clarify whether combinatorial therapy should target multiple pathways within a single biological state or within different niches within heterogeneous tumors.

Finally, retrospective studies of large, annotated patient cohorts, such as TCGA, could assess whether combination signatures, such as, for instance, a Notch/Wnt/Neuroactive program, are prognostic for relapses or predictive of TMZ response. Prospective clinical studies will be essential for integrating these indicators into precision medicine techniques, allowing for the classification of individuals for carefully designed, developed combinatorial regimens aimed at overcoming CSC-mediated resistance.

## Supplementary Information

Below is the link to the electronic supplementary material.

Supplementary Material 1

Supplementary Material 2

Supplementary Material 3

Supplementary Material 4

## Data Availability

This RNA-Seq project has been deposited at DDBJ Sequence Read Archive under accession numbers SRR35841117-SRR35841125 (BioProject: PRJNA1345671, BioSample accession: SAMN52754693-SAMN52754701). The quality of the raw readings was evaluated using FastQC v0.12.1.

## Abbreviations

ABC: ATP-binding cassette
ADCY: Adenylyl cyclase
CACNA1G/H/D: Calcium voltage-gated channel subunit alpha 1G/H/D subunits
CDK18: Cyclin dependent kinase 18
CDKL5: Cyclin-dependent kinase-like 5
CDH5: Cadherin 5
CNTN2: Contactin-2
CHRM1: Cholinergic receptor muscarinic 1
CHRM4: Muscarinic acetylcholine receptor M4
COL21A1: Collagen type XXI alpha 1
COL4A5: Collagen type IV alpha 5 chain
COL1A2: Collagen type I alpha 2
DLL1: Delta like canonical Notch ligand 1
DNER: Delta and Notch-like epidermal growth factor-related receptor
DRD2: Dopamine receptor D2
ECM: Extracellular Matrix
EDN3: Endothelin 3
EPHA10: Ephrin receptor A10
EPHB1: Ephrin type-B receptor 1
ERBB3: Receptor tyrosine-protein kinase erbB-3
FAK: Focal adhesion kinase
FGFR2/3: Fibroblast growth factor receptors 2/3
FN1: Fibronectin1
FXYD5: Dysadherin
GABRA4: Gamma-aminobutyric acid receptor subunit alpha-4
GABRB1: Gamma-aminobutyric acid receptor subunit beta-1
GABRR2: Gamma-aminobutyric acid receptor subunit rho-2
GRIA2/4v: Glutamate ionotropic receptor AMPA type subunit 2/4
GRIK5: Glutamate receptor, ionotropic kainate 5
GRIN1: Glutamate ionotropic receptor NMDA type subunit
GRID2: Glutamate ionotropic receptor delta type subunit 2
HES: Hes family bHLH transcription factor
HOX: Homeobox gene
HTR6: 5-hydroxytryptamine receptor 6
ID1/2: Inhibitor of DNA binding 1/2
IGF2: Insulin-like growth factor 2
ITGA3/5: Integrin subunit alpha 3/5
LEF1: Lymphoid enhancer factor 1
KCNB1: Potassium voltage-gated channel subfamily B member 1
KCNN1: potassium calcium-activated channel subfamily N member 1
KCNQ4: Potassium voltage-gated channel subfamily Q member 4
MLH1: DNA mismatch repair protein Mlh1
NICD1: Notch intracellular cleaved domain 1
NPY2R: Neuropeptide Y receptor Y2
NTRK2: Neurotrophic receptor tyrosine kinase 2, TrkB
NTRK3: Neurotrophic receptor tyrosine kinase 3, TrkC
PDGFB: Platelet derived growth factor subunit B
PCK1: Phosphoenolpyruvate carboxykinase 1
ROBO2: Roundabout guidance receptor 2
SGK1: Serum/glucocorticoid regulated kinase 1
SLC17A7: Vesicular glutamate transporter 1
SEMAs: Semaphorins
SHANK1: SH3 and multiple ankyrin repeat domains protein 1
SOCS2/3: Suppressor of cytokine signaling 2/3
SOX: SRY-related HMG-box gene
SSTR2/3: Somatostatin receptor type 2/3
SUR1: Sulfonylurea receptor 1
UNC5A/B: Netrin receptors A/B
VCAM1: Vascular cell adhesion molecule 1
VGF: VGF nerve growth factor inducible
VIPR1: Vasoactive intestinal polypeptide receptor 1
TP73: Tumor protein p73
YAP1: Yes associated protein 1
WNT: Wingless and Int-1
