# Supplementary material for: Distinct molecular pathways leading to dosage-dependent temozolomide resistance in GBM stem cells
Source: Cancer Cell Int. 2026 Feb 24;26:118. doi: 10.1186/s12935-026-04213-6 (PMC12961842; doi:10.1186/s12935-026-04213-6)
Supplement: Supplementary file 4 — Supplementary Material 4 [file 12935_2026_4213_MOESM4_ESM.docx]

| **GENE** | **DMSO** | **2.5 μM TMZ** | **12.5 μM TMZ** |
| --- | --- | --- | --- |
| **EGFR** | 100 ± 10 | 106.5 ± 8.5 n=2 | 101.1 ± 6.100 n=2 |
| **NICD1** | 100 ± 10 | 112.7 ± 17.6 n=2 | 404.0 ± 19.0 n=2** |
| **HES-1** | 100 ± 10 | 122.5 ± 12.5 n=2 | 195.0 ± 7.5 n=2* |
| **BCL-2** | 100 ± 10 | 98.0 ± 3.0 n=2 | 388.7 ± 23.1 n=2* |
| **Survivin** | 100 ± 10 | 92.0 ± 6.0 n=2 | 195.4 ± 11.7 n=2* |
| **AKT1** | 100 ± 10 | 98.5 ± 8.5 n=2 | 105.5 ± 3.5 n=2 |
| **pAKT1 (S473)** | 100 ± 10 | 108.1 ± 3.6 n=2 | 183.0 ± 12.0 n=2 |
| **ERK 1/2** | 100 ± 10 | 103.7 ± 13.7 n=2 | 106.0 ± 3.2 n=2 |
| **pERK (Thr202/Tyr204)** | 100 ± 10 | 415.2 ± 10.2 n=2** | 181.5 ± 11.5 n=2* |
| **β-Actin** | 100 ± 10 | 94.0 ± 3.6 n=2 | 103.8 ± 3.6 n=2 |
